# Supplementary material for: Evaluating the impact of a common elements-based intervention to improve maternal psychological well-being and mother–infant interaction in rural Pakistan: study protocol for a randomised controlled trial
Source: BMJ Open. 2021 Jul 7;11(7):e047609. doi: 10.1136/bmjopen-2020-047609 (PMC8264893; doi:10.1136/bmjopen-2020-047609)
Supplement: Author's manuscript [file bmjopen-2020-047609.draft_revisions.pdf]

BMJ Open is committed to open peer review. As part of this commitment we make the peer review history of every article we publish publicly available.

When an article is published we post the peer reviewers' comments and the authors' responses online. We also post the versions of the paper that were used during peer review. These are the versions that the peer review comments apply to.

The versions of the paper that follow are the versions that were submitted during the peer review process. They are not the versions of record or the final published versions. They should not be cited or distributed as the published version of this manuscript.

BMJ Open is an open access journal and the full, final, typeset and author-corrected version of record of the manuscript is available on our site with no access controls, subscription charges or pay-per-view fees (http://bmjopen.bmj.com).

If you have any questions on BMJ Open's open peer review process please email info.bmjopen@bmj.com

BMJ Open

Evaluating the impact of a common elements based intervention to improve the maternal psychological wellbeing and mother-infant interaction in rural Pakistan: Study protocol for a randomized controlled trial

Journal:	*BMJ Open*
Manuscript ID	bmjopen-2020-047609
Article Type:	Protocol
Date Submitted by the Author:	03-Dec-2020

Complete List of Authors:	Huma, Zill-e-; Human Development Research Foundation, Implementation Science; University of Liverpool Faculty of Health and Life Sciences, Department of Primary Care and Mental Health<br>Gillani, Ayella; Human Development Research Foundation, Implementation Science<br>Shafique, Fakhira; Human Development Research Foundation, Implementation Science<br>Rashid, Alina; Human Development Research Foundation, Implementation Science<br>Mahjabeen, Bushra; Human Development Research Foundation, Implementation Science<br>Wang, Duolao; Liverpool School of Tropical Medicine, Department of Clinical Sciences<br>Rahman, Atif; University of Liverpool Faculty of Health and Life Sciences, Department of Primary Care and Mental Health<br>Hamdani, Syed Usman; University of Liverpool Faculty of Health and Life Sciences, Department of Primary Care and Mental Health ; Human Development Research Foundation, Implementation Science

Keywords:	MENTAL HEALTH, Community child health < PAEDIATRICS, Depression & mood disorders < PSYCHIATRY, Public health < INFECTIOUS DISEASES, Developmental neurology & neurodisability < PAEDIATRICS, Anxiety disorders < PSYCHIATRY

SCHOLARONE™ Manuscripts

I, the Submitting Author has the right to grant and does grant on behalf of all authors of the Work (as defined in the below author licence), an exclusive licence and/or a non-exclusive licence for contributions from authors who are: i) UK Crown employees; ii) where BMJ has agreed a CC-BY licence shall apply, and/or iii) in accordance with the terms applicable for US Federal Government officers or employees acting as part of their official duties; on a worldwide, perpetual, irrevocable, royalty-free basis to BMJ Publishing Group Ltd ("BMJ") its licensees and where the relevant Journal is co-owned by BMJ to the co-owners of the Journal, to publish the Work in this journal and any other BMJ products and to exploit all rights, as set out in our licence.

The Submitting Author accepts and understands that any supply made under these terms is made by BMJ to the Submitting Author unless you are acting as an employee on behalf of your employer or a postgraduate student of an affiliated institution which is paying any applicable article publishing charge ("APC") for Open Access articles. Where the Submitting Author wishes to make the Work available on an Open Access basis (and intends to pay the relevant APC), the terms of reuse of such Open Access shall be governed by a Creative Commons licence – details of these licences and which Creative Commons licence will apply to this Work are set out in our licence referred to above.

Other than as permitted in any relevant BMJ Author's Self Archiving Policies, I confirm this Work has not been accepted for publication elsewhere, is not being considered for publication elsewhere and does not duplicate material already published. I confirm all authors consent to publication of this Work and authorise the granting of this licence.

For peer review only

Evaluating the impact of a common elements based intervention to improve the maternal psychological wellbeing and mother-infant interaction in rural Pakistan: Study protocol for a randomized controlled trial

Zill-e-Huma [1, 2], Ayella Gillani [1], Fakhira Shafique [1], Alina Rashid [1], Bushra Mahjabeen [1]
Duolao Wang [3], Atif Rahman [2], Syed Usman Hamdani [1, 2]

[1]Human Development Research Foundation, Rawalpindi, Pakistan

[2]Department of Primary Care and Mental Health, University of Liverpool, Liverpool, United Kingdom

[3]Liverpool School of Tropical Medicine, Liverpool, United Kingdom

Corresponding Author:
Zill-e-Huma
Human Development Research Foundation (HDRF), Pakistan
E: zille.huma@hdrfoundation.org

For peer review only

For peer review only - http://bmjopen.bmj.com/site/about/guidelines.xhtml

Abstract

Introduction Millions of children in low resource settings are at high risk of poor development due to factors such as under nutrition, inadequate stimulation and maternal depression. Evidence based interventions to address these risk factors exist, but often as a separate and overlapping packages. The current study aims to evaluate the effectiveness of a common elements-based intervention to improve mother-infant interaction at 12-months post-partum.

Method and analysis A two arm, single blind, individual randomized controlled trial (RCT) is being carried out in the community settings of the rural sub-district of Gujar Khan in Rawalpindi, Pakistan. 250 pregnant women in third trimester with distress (Self-Reporting Questionnaire (SRQ), cut-off score >9) have been randomized on 1:1 allocation ratio into intervention (n=125) and Treatment as Usual (TAU) arms (n=125). The participants in the intervention arm will receive 15 individual sessions of intervention on a monthly basis by non-specialist facilitators. The intervention involves components of early stimulation, learning through play, responsive feeding, guided discovery using pictures, behavioural activation and problem solving. The primary outcome is caregiver-infant interaction at 12-months post-partum. The secondary outcomes include maternal psychological wellbeing, quality of life, social support and empowerment. Infant secondary outcomes include growth, nutrition and development. The data will be collected at baseline, 6- and 12-months' post-partum. A qualitative process evaluation will be conducted to inform the feasibility of intervention delivery.

Ethics and dissemination Ethics approval for the present study was obtained from the Human Development Research Foundation (HDRF) Institutional Review Board, Islamabad Pakistan. The findings of the present study will be published in peer-reviewed journals and presented at conferences and community forums.

Trial registration: Registered prospectively with ClinicalTrials.gov as "a common elements-based intervention to improve maternal psychological well-being and mother-infant interaction". Identifier: NCT04252807

Keywords: Maternal distress; mother-infant interaction; common elements treatment approach; nurturing care; low resource settings

For peer review only

Article summary

- To address the burden associated with poor infant development and perinatal distress we have developed a common elements based early intervention. The intervention is designed to be delivered through non-specialist facilitators in low resource community settings.
- In a single blind, individual randomised controlled trial, we will evaluate the impact of intervention using a pragmatic primary outcome measure i.e. 5-minute observation of a live caregiver-child interaction.
- The COVID-19 pandemic has resulted in limited face to face contact with the study participants and the intervention is being delivered using blended methodology (telephonic delivery as well as individual sessions where possible). We shall evaluate the feasibility

For peer review only - http://bmjopen.bmj.com/site/about/guidelines.xhtml

and acceptability of blended intervention delivery methodology using mixed methods process evaluation.

- Continued participation of mother-infant dyads through the completion of the intervention and to the final assessment point at 12-months postpartum could be a challenge in the COVID-19 pandemic.
- Exclusion of women with perinatal depression, may limit the generalizability of findings.

Introduction

In South Asia, 35-45% children are undernourished [1] and over 40 million lag behind in their cognitive and socio-emotional development [2]. The situation is compounded by maternal depression, which affects over a quarter of all women [3]. Suboptimal functioning and agency in depressed mothers is a barrier to implementation of any intervention directed at the young child [4]. Together, these factors have a negative impact on provision of nurturing care that is essential for optimal development [5-8].

Pakistan has a population of about 200 million people, of which three quarters live in rural areas. 47% of children under 5 years of age suffer from stunted growth and development in Pakistan [9]. By conservative estimate about eight million children do not achieve their full cognitive potential [2]. Prevalence studies in the proposed study area have reported high rates of perinatal depression in mothers ranging from 25-37% and a strong and independent association of maternal depression with low birth weight and under nutrition [10]. Lack of early interventions mean that many of these children might develop cognitive, social and emotional difficulties, contributing further to emotional and financial stress in the families [11]. Considerable effort has gone into child survival programmes, leading to a reduction in child mortality rates over the last decade. However, improved child survival means there are many more children who are at risk of developmental delay. Evidence based interventions to address risk factors for poor child development exist, but often as separate and overlapping packages of interventions.

To address these challenges, we have developed a package of intervention using a common elements approach [12] that combines evidence based elements from packages of care addressing early infant stimulation, responsive feeding and maternal distress [13-15]. These approaches recognize the importance of early critical years and role of the primary caregiver – normally the mother to provide nutrition and an optimal environment for interaction and stimulation. The mother, in turn, requires psychosocial support, education and skills training specific to Early Child Development (ECD), provided via a well monitored and supervised delivery system.

We aim to evaluate the effectiveness of common elements-based intervention compared to usual care to improve mother-infant interaction along with improvements in maternal psychological wellbeing, infant growth, nutrition and development at 12-months post-partum in low resource rural community settings of Pakistan.

Hypotheses

We hypothesize that common element based integrated intervention will result in improvements of at-least 0.4 effect size in caregiver-child interaction measured using Observation of Mother and Child Interaction (OMCI) in intervention arm compared with the Treatment –As-Usual in control arm, at 12 months' postpartum. Our secondary hypotheses (exploratory) are that our intervention will result in improvement in infant growth, nutrition and development and will improve maternal psychological wellbeing and quality of life.

Methods and analysis

Study design

For peer review only - http://bmjopen.bmj.com/site/about/guidelines.xhtml

A two-arm, single blind, individual randomized controlled trial with mixed-methods process monitoring and evaluation is being carried out to evaluate the effectiveness of intervention. The participants are recruited from a rural sub-district, randomized into intervention and control arms, on a 1:1 allocation ratio.

Patient and public involvement

The multicomponent intervention is based on existing community programmes developed by the research team working collaboratively with the people from local communities including mothers with post-natal depression, fathers, extended family members, community health workers and local volunteers [4, 6, 11, 16]. The relevant stakeholders (community representatives and gate keepers and mental health experts) were involved through consultative workshops (Theory of Change workshops) in the formative research to develop a hypothesized pathway for the implementation of intervention in the study sub-district. The findings of the present study will be disseminated to participants through presentation at community forums.

Study settings

The present study is being conducted in sub-district of Gujar Khan in Rawalpindi Pakistan. The sub-district represents a typical rural area of Pakistan and have poor demographic and health profile indicators (with high fertility rate – 4 births per woman and low female literacy rate i.e. 45%) [17], where 1 in every 4 women suffer from perinatal depression [17, 18]. The infant mortality rate in Rawalpindi has been estimated to be 55 per thousand live births, the under-5 mortality rate is 82 per thousand live births and the percentage of underweight children below 5 years of age is 25% [19]. Primary health care is delivered through a network of Basic Health Units (BHUs), each providing care to about 15,000–20,000 people. Each BHU is staffed by a doctor, midwife, vaccinator, and 15–20 village-based Community Health Workers (CHWs) also called Lady Health Workers (LHWs). These women have completed secondary school and are trained to provide preventive maternal and child health care, and education in the community. Each LHW is responsible for about 100 households in her village. The participants shall be recruited from the catchment areas of LHWs.

Research participants:

The target population for the present study is pregnant women in third trimester with distress (screen positive on Self-Reporting Questionnaire, cut-off score ≥ 9).

Eligibility criteria of participants

Inclusion criteria:

- Pregnant with third trimester (28 gestational week)
- Age 18-40 years
- Intend to reside in the study areas until the completion of the study
- Score ≥9 on the SRQ

Exclusion criteria

- Women who require immediate or on-going medical or psychiatric care reported.
- Severe previous or current obstetric morbidity including eclampsia and antepartum hemorrhage;
- Medical disorders that require inpatient management (e.g., diabetes, hypertension, thromboembolism, cardiac disease)

For peer review only - http://bmjopen.bmj.com/site/about/guidelines.xhtml

Sample size calculations

The power calculations are based on one primary outcome, i.e. mother-child interaction on Observation of Mother and Child Interaction (OMCI) tool [20] at 12 months' post-partum. The scoring of tool is based on the frequency of the occurrence of responsive behaviours of mother with higher scores indicating more responsive interactions. Studies from Pakistan reported an effect size of 0.4 on OMCI as a result of responsive stimulation intervention [8]. We therefore assume an effect size of 0.4 on primary outcome i.e. caregiver-child interaction measured using OMCI at 12 months' post-partum. Assuming an effect size of 0.4, with 80% power, 0.05 significance, and a two-sided hypothesis test and accounting for 20% attrition, we will need 250 caregiver-child dyads (125 in each arm).

Recruitment procedure

Before recruiting participants in the trial, the local community stakeholders were taken on-board. 250 pregnant women in their third trimester, who fulfilled the eligibility criteria, are being recruited from the catchment areas of LHWs, working in the sub-district of Gujar Khan. Written informed consent is obtained from the study participants by trained assessment team. A complete record of assessment against eligibility criteria, and reasons for refusal is maintained for those who do not consent for participation in the trial.

All potential eligible pregnant women in third trimester are screened for psychological distress by research team using Self-Reporting Questionnaire (SRQ). Psychological distress is represented by subscales of depression/anxiety, somatic symptoms, reduced vital energy, and depressive thoughts during the past month. The scale reflects multidimensional nature of mental illnesses. It has 20 items scored on 0 or 1. A score of 1 indicates that the symptom was present during the past month; a score of 0 indicates that the symptom was absent. The maximum score is therefore 20, with a higher score indicating higher psychological distress. A score of ≥ 9 indicates risk of postnatal depression [8] and mothers with these scores have been recruited in the study.

Intervention

The intervention called 'Nurturing Care' combines common elements from evidence based interventions for maternal psychosocial distress (e.g. WHO Thinking Healthy Program [15]), early stimulation and responsive feeding (e.g. UNICEF Care for Child Development Package [13]), and child care (e.g. WHO Caregivers Skills Training program [14]) for optimal development of at risk mother and children.

The intervention package aims to improve a) mother psychological distress by promoting problem solving, developing empathic listening, improving family support, guided discovery using pictures, and behavioural activation; b) child development through psycho-education, responsive breastfeeding and c) mother-infant interaction through early stimulation and learning through play. The intervention is designed to be delivered by non-specialist facilitators in 15 monthly sessions in community settings.

**Description of intervention sessions**			
**Sessions**	**Content**	**Timing**	**Recipients**
1: Introduction and Rapport building	- Introducing program and engaging mother and her family<br>- Developing empathetic relationship with mother	Beginning of 7th month of pregnancy	Mother and family members

For peer review only - http://bmjopen.bmj.com/site/about/guidelines.xhtml

	- Ensuring family support system		
2: Maternal wellbeing	- Assessment of psychosocial problems of mother<br>- Use of guided discovery and behavioral activation for improving mother's health (strategies for improving overall well-being, including diet, rest, relaxation and sleep)	7th month of pregnancy	Mother and family members
3: Improving mother relationship with significant others	- Thought challenging and behavior activation using guided discovery (strategies for improving interpersonal relationships and mobilizing social support)<br>- Awareness about complication during pregnancy	8th month of pregnancy	Mother and family members
4: Improving mother relationship with infant during pregnancy	- Thought challenging and behavior activation using guided discovery (strategies for improving bond with the infant during pregnancy. knowledge and planning of safe delivery and importance of colostrum)	9th month of pregnancy	Mother
4: Follow-up session after delivery	- Following up after child birth<br>- Psycho-education about child development	Immediate post-delivery	Mother
5: Improving child's physical health	- Thought challenging and behavior activation using guided discovery (strategies for awareness on benefits of exclusive breastfeeding)<br>- Responsive breast feeding<br>- Addressing the concept of child crying because of insufficient milk<br>- Increasing breast milk by diet and improved frequency- addressing insufficiency of milk<br>- Family support for breast feeding<br>- Assessment of post-partum psychosocial problems of mother	8 weeks postnatal	Mother

For peer review only - http://bmjopen.bmj.com/site/about/guidelines.xhtml

5A: Improving maternal health	- Assessment of psychosocial problems of mother<br>- Use of guided discovery and behavioral activation for improving mother's health	9 weeks postnatal	Mother
5B: Improving maternal health	- Use of guided discovery and behavioral activation for improving mother's relationship with others	10 weeks postnatal	Mother
5C: Improving maternal health	- Use of guided discovery and behavioral activation for improving mother's relationship with child	11 weeks postnatal	Mother
6: Importance of mother child interaction	- Exclusive Breast Feeding (EBF) for 6months<br>- Birth spacing<br>- Suitable environment- birth spacing<br>- Interaction, love and care for child<br>- Selecting play activity and conducting with mother and child	12 weeks postnatal	Mother and infant
7: Follow up on early stimulation activities	- Revision of activities discussed in session 6<br>- Importance of vaccination	16 weeks postnatal	Mother and Child
8: Awareness on birth spacing; improving child's health	Thought challenging and behavior activation using guided discovery (strategies for awareness on birth spacing and its relation to child's health)<br><br>- Exclusive breast feeding for 6-month<br>- Birth spacing<br>- Suitable environment- birth spacing<br>- Modern methods of contraception<br>- Interaction, love and care for child<br><br>Selecting play activity and conducting with mother and child	20 weeks postnatal	Mother

For peer review only - http://bmjopen.bmj.com/site/about/guidelines.xhtml

9: Introduction to solid food	- Interaction and play with child<br>- Selecting play activity and conducting with mother and child<br>- Introduction to Solid food and concept of super food<br>- Food and hygiene<br>- Continuation of breastfeeding with weaning at 6m<br>- Importance of vaccination	24 weeks postnatal	Mother
10: Introduction to mother child interaction. Improving mother child interaction through responsiveness and praise	- Skill based activities to promote mother-child interaction<br>- Homework charts are given to monitor the activity.<br>- Learning through play activities are reinforced<br>-	28 weeks postnatal	Mother and infant
11: Importance of following child's interest	- Skill based activities to promote mother-child interaction<br>- Homework charts are given to monitor the activity.<br>- Learning through play activities are reinforced	32 weeks postnatal	Mother and infant
12: Importance of following child's lead	- Skill based activities to promote mother-child interaction<br>- Homework charts are given to monitor the activity.<br>- Learning through play activities are reinforced	36 weeks postnatal	Mother and infant
13: Promote child language development	- Skill based activities to promote mother-child interaction<br>- Homework charts are given to monitor the activity.<br>- Learning through play activities are reinforced	40 weeks postnatal	Mother and infant
14: Scaffolding	- Skill based activities to promote mother-child interaction<br>- Homework charts are given to monitor the activity.<br>- Learning through play activities are reinforced	44 weeks postnatal	Mother and infant

For peer review only

For peer review only - http://bmjopen.bmj.com/site/about/guidelines.xhtml

15: Review of all 5 strategies	Revision of all strategies and next line of action. Learning through play activities are reinforced	48 weeks postnatal	Mother and infant

Training and supervision of non-specialist facilitators in 'Nurturing Care' intervention

The delivery agents of the 'Nurturing Care' intervention are female non-specialist facilitators who are graduates in psychology/social sciences with little or no prior experience of delivering psychosocial interventions to mothers with perinatal distress and their new-born infants and have achieved competency to deliver 'Nurturing Care' intervention following training and practice sessions. These non-specialist facilitators were selected from the universities in the study sub-district through a job advertisement and interviews.

A cascade model of training and supervision has been followed to deliver 'Nurturing Care' intervention. The training consisted of classroom teaching of basic theoretical concepts of 'Nurturing Care' intervention, its strategies, group discussions and role-plays. The program trainers trained 15 non-specialist facilitators. The training consisted of 10-days class-room training followed by practice cases under the supervision of program trainer. The participants of this practice group were pregnant women without distress and their infants, selected from the same community after seeking informed consent for participation in the training. The program trainer performed live competency rating during practice cases using an adapted version of ENhancing Assessment of Common Therapeutic factors (ENACT) tool for 'Nurturing Care' intervention (Kohrt et al. 2015). Only competent non-specialist facilitators (mean score above 2.5 on all domains of adapted ENACT) have been allowed to deliver 'Nurturing Care' intervention. Each non-specialist facilitator has been assigned a case load of 8-10 mothers to deliver intervention. The fidelity of the intervention is being evaluated using an adapted ENACT tool developed for the program. 20% of randomly selected intervention sessions from each non-specialist facilitators at different stages of the intervention delivery will be rated by the program trainers to assess the fidelity of intervention delivery by non-specialist facilitators.

Supervision

Program trainers are experts in perinatal mental health and infant development and provide weekly supervision to non-specialist facilitators to ensure intervention fidelity. Supervision involves discussion of difficulties encountered in delivering intervention delivery and, as well as self-care of the non-specialist facilitators.

Treatment as Usual (TAU)

As a part of treatment-as-usual, participants in both study arms receive routine visits by the Lady Health Workers (LHWs) of their respective areas. The LHWs are trained to provide preventive maternal and child care including psycho-education about family planning and maternal and child health; provide free of cost contraceptives and antenatal-iron-folic acid supplements throughout the pregnancy starting from second trimester via door to door visits to the households of their allocated areas. In coordination with traditional birth attendants and affiliated nearest health facility, LHWs also provide referral service to mothers for safe motherhood.

If, during the study's assessments time-points participants in TAU arm show severe psychiatric disorders that require immediate specialist treatment and follow-up, they will be referred to tertiary mental health care facility, Institute of Psychiatry, Benazir Bhutto Hospital, Rawalpindi. A complete record of services received by trial participants in both arms will be made using the Client Services Receipt Inventory (CSRI).

For peer review only - http://bmjopen.bmj.com/site/about/guidelines.xhtml

Outcomes measures

The effectiveness of intervention will be evaluated on a range of caregiver and child outcomes. The outcome measures will be administered by the trained assessment team blind to the allocation status of participants at baseline, 6 and 12-months' postpartum. The detail of the outcome measures is given below (see Table 2 for schedule of assessments);

Primary Outcome

Caregivers Child Interaction (CCI): Maternal responsive caregiving behaviours will be assessed at 12-months' postpartum across two arms (primary endpoint) using the **Observation of Mother and Child Interaction (OMCI) tool [20].** OMCI tool is based on responsive parenting framework proposed by Landry and colleagues [21]. The research assistants will observe a live 5-minute mother-infant interaction while mother and infant will play together with a picture book. The tool comprises of 19 items covering maternal affect, maternal touch, maternal verbalization, sensitivity and contingent responses, scaffolding, language stimulation, focus, child affect, child focus, child's communication efforts, and mutual enjoyment. The scoring format is based on the frequency of the occurrence of behaviours with higher scores indicating more responsive interactions. The OMCI tool has been validated for use in similar population in Pakistan [20].

Infant secondary outcomes

1. **Bayley's Scales of Infant Development (BSID III**) [22] will be used to measure cognitive, language, motor, socio-emotional development and adaptive behaviour at 12-months' postpartum.
2. **Ages and Stages Questionnaire 3rd Edition (ASQ3) [23]** will be used to measure developmental delays in communication, gross motor, fine motor, problem solving, and personal social domains at 12-months' postpartum. It provides cut-off score in 5 domains of development that indicates possible need for further evaluation. It highlights results that fall in a "monitoring zone," to make it easier to keep track of children at-risk.
3. Home environment measured by **Home inventory [24]:** Quality and quantity of stimulation and support in the child's home will be measured using home inventory at 12-months' postpartum.
4. **Anthropometric measurements [25]**: Infants in both groups will be weighed and measured with standard techniques at 12-months' postpartum. Growth data will be converted into SDs (Z scores) for weight and length with Epi Info 2002 (version 3.4.1).
5. **Early breastfeeding (EBF) [26]**: Assessors will document what the infant was fed in the last 24 hours at 6 months postpartum. This information will be categorized as either EBF, partial breastfeeding (giving an infant some breastfeeding, and some artificial foods, either milk, cereal, or other food items), or no breastfeeding. If the infant will not exclusively breastfeed, details of when (age of infant) and why EBF was discontinued will be recorded. At the first 6-months follow-up, details about early initiation, discarding colostrum, use of pre-lacteals, and reasons for delaying or not initiating breastfeeding will be assessed.
6. The number of **diarrhoeal episodes** in the infants during the 2 weeks before interview will be recorded at 6 and 12-months' postpartum, with a questionnaire used in previous studies [27]. Diarrhoea will be defined as three or more unformed stools passed in 24 hours, and a diarrhoeal episode will be defined as being separated from another episode by at least 3 diarrhoea-free days.

For peer review only - http://bmjopen.bmj.com/site/about/guidelines.xhtml

7. Records of **immunisation** will be assessed for all infants in the study, and infants will be classified as those with or without up-to-date immunisation status 6 and 12-months' postpartum.

Maternal secondary outcomes;

8. Maternal distress will be measured by **the Self Reporting Questionnaire (SRQ**) [28] at baseline, 6 and 12-months postpartum. SRQ is a 20-item self-report measure to detect non-specific psychological distress, developed by the World Health Organization (WHO). Psychological distress is represented by subscales of depression/anxiety, somatic symptoms, reduced vital energy and depressive thoughts. The SRQ items are scored 0 or 1. A score of 1 indicates the presence of symptoms of psychological distress during past month and a score of 0 indicates absence of symptoms. The maximum score indicates presence of higher psychological distress.
9. Caregivers' health related quality of life will be measured by **Paediatric Quality of Life Inventory (PedsQL)** family impact module [29] at baseline and 12-months postpartum. The PedsQL is 36 item impact module scale that encompasses 6 sub-scales measuring parent self-reported functioning. These subscales measure physical functioning, emotional functioning, social functioning, daily activities and family relationships. Items are rated on a 5-point Likert scale (0 = *never* to 4 = *almost always*).
10. The level of social support will be measured using **Multi-Dimensional Scale of Perceived Social Support (MSPSS) [30]** will be measured baseline and 12-months' postpartum. It includes 12 items which cover three dimensions: support from family, friends and significant other. Each item is rated on a 7-point Likert-scale (1 = *very strongly disagree*; 7 = *very strongly agree*). A total score is calculated by summing the responses of all items (range 12–84) with higher scores indicating higher levels of perceived social support.
11. Caregiver's level of functioning will be measured using **World Health Organization Disability Assessment Schedule (WHODAS) [31]** at baseline and 12-months' post-partum. WHODAS assesses participants' health-related difficulties in the level of functioning in six domains of life (understanding and communicating; moving and getting around; attending to one's hygiene, dressing, eating and staying alone; interacting with other people; domestic responsibilities, leisure, work and school and joining in community activities, participating in society) over the past 30 days.
12. **Maternal Financial empowerment**: Women's empowerment will be measured at baseline and 12-months postpartum, using a questionnaire consisting of: (a) two items previously used in the same population [32] that assess financial empowerment (i.e., whether a woman is given a lump-sum of money for day-to-day expenses and whether she can make independent decisions about its use) and (b) 7 items [33] to measure relative freedom from domination within the family (i.e., whether in the past year family had taken money, land, jewellery or livestock from her against her will, prevented her from visiting her parental home, or prevented her from working outside the home) and economic security and contribution (i.e., does she possess any land, productive assets, and or cash). Women who answer '*yes*' to both items on financial empowerment and three items from economic security and contribution subscales and '*no*' to all items regarding family domination will be classified as 'empowered'.
13. Utilization of health care will be measured by **Client Service Receipt Inventory (CSRI)** adapted for perinatal population of Pakistan at 12 months postpartum [26].

For peer review only - http://bmjopen.bmj.com/site/about/guidelines.xhtml

Table 2: Schedule of assessments

Sr. no.	Outcomes		Time points		
		Screening	Baseline 3rd trimester of pregnancy	6-months post-partum	12 months postpartum
Primary outcome					
1.	OMCI				X
Secondary outcomes					
2.	SRQ	X	X	X	X
3.	WHO-QoL		X		X
4.	Maternal empowerment-brief questionnaire		X		X
5.	BSID-III				X
6.	ASQ3				X
7.	Home inventory				X
8.	Weight and height measurements				X
9.	Early breastfeeding-brief questionnaire			X	
10.	Diarrhoeal episodes -brief questionnaire			X	X
11.	Immunization-record form			X	X
12.	CSRI				X

OMCI, Observation of Mother-Child Interaction; SRQ, Self-reporting Questionnaire; WHO-QoL, World Health Organization, Quality of Life; BSID-III, Brief questionnaire, Bayley Scale of Infant Development-III; ASQ3, Ages and Stages Questionnaire-3; CSRI, Client Service Receipt Inventory

Randomization and blinding

The participants were randomized into intervention or control arm on a 1:1 basis using computerized software by an independent researcher. The allocation concealment was ensured by keeping random sequence in sequentially numbered, opaque, sealed envelopes, at the off-site centre. Only assessment team (conducting baseline and follow-up assessment) will be blind to the treatment allocation of trial participants. To maintain the blinding, all trial participants will be briefed, prior to the assessment about the significance of blinding and the importance of not disclosing their allocation status to the assessment team members. If un-blinding does occur, this will be documented, the assessment will be halted and a new assessor will be assigned to complete the assessment.

Ethical considerations

Ethical approval for the study was obtained from Institutional Review Board (IRB) of Human Development Research Foundation (HDRF). Informed consent was obtained from all participants prior to data collection. Privacy and confidentiality of the research participants will be ensured throughout the study.

Adverse events reporting

For peer review only - http://bmjopen.bmj.com/site/about/guidelines.xhtml

All adverse events (AEs) and serious adverse events (SAEs) reported by the trial participants, their husband and in-laws of recruited participants, LHWs or observed by the investigator or the research team (assessment & intervention team) will be recorded by data manager. AE is defined as any undesirable experience occurring to a subject during the time frame of the study, whether or not considered relevant to the research procedure. All AEs will be collected by assessment team at 12-months' postpartum follows and will be reviewed periodically by trial steering committee which will determine the necessary steps to be taken with respect to the ongoing trial.

Data management

The baseline and follow-up assessments data will be collected by the assessment team on android based application using hand held devices (tablets). Data will be stored at the secure web server and will be checked for consistency & quality at the end of each day. All data sets will be stored in password protected computer, only accessible by the responsible individual. The data collected in hard copies (collected by the interviewers) will be safely stored in locked cabinets. Qualitative data will be fully anonymized and coded. No identifying names or details will be recorded.

Statistical analysis

The findings will be reported according to the CONSORT guidelines for RCT [34] (Figure 1). This will include the flow of research participants through each stage of the trial, including the number eligible, randomly assigned, receiving the intended treatment, completing the study protocol and analysed for the primary outcome. The primary focus of analyses will be on intent-to-treat analyses consisting of all participants included according to the arms in which they are randomized and secondary analyses will be based on per-protocol population.

For the analysis of the primary outcome (caregiver-child interaction across two arms at 12-months post-partum), a linear mixed model will be employed with treatment, visit, interaction between treatment and visit as fixed effect, the baseline value of the mothers' psychosocial distress as covariate, and subject as random effect. In addition, adjusted linear mixed model analysis will be performed with the pre-specified covariates (gender of infant, socio-economic status and mother's education) measured at baseline being added into the above mixed model, which will be identified and listed in the statistical analysis plan. The crude and adjusted mean differences in the primary outcome together with its 95% confidence intervals at 12-months will be derived from the mixed models. In addition, subgroup analysis of primary endpoint will be performed on the above pre-specified covariates.

Analysis of secondary continuous outcomes with single follow-up measurement will be done in a similar fashion as the primary endpoint analysis. Analysis of secondary continuous outcomes with repeated follow-up measurement will be performed using a mixed-model with treatment visit, interaction between treatment and visit as fixed effects, the baseline value of the outcome as covariate, and subject as random effects. The analysis of categorical outcomes will also use a generalized linear mixed model with treatment as fixed effect, baseline measurement of primary endpoint as covariate and subject as random effect. Odds ratios with their 95% confidence intervals will be derived from the generalized linear mixed model analysis of a categorical outcome. Sensitivity analysis will be performed on the covariates with missing values imputed [35]. Missing data will be treated as missing at random in the mixed model analysis and no imputation of primary and secondary endpoints will be made. To assess the sensitivity of the result to this assumption, the last observation carried forward strategy will be used to compute missing primary and secondary endpoints.

For peer review only - http://bmjopen.bmj.com/site/about/guidelines.xhtml

Potential mediators

We will explore pathways through which the intervention may influence child out-comes. We focus on maternal wellbeing and responsiveness which we define broadly to include the following variables: *Maternal Responsiveness*; there is strong empirical evidence that the quality of the mother-child interactions, such as her sensitivity and responsiveness, is associated with both maternal depression and child development [36-39]. There is also some evidence that perinatal depression interventions, especially ones that include a parenting component, can improve maternal responsiveness, resulting in improved developmental outcomes [40]. We will assess maternal responsiveness with the HOME Inventory, an extensively used measure of the child's family environment [41]. The HOME components, including responsiveness, have been linked with multiple developmental outcomes [42, 43] have been used to evaluate the impact of maternal interventions [44]. *Social support*: Low social support is a consistent predictor of mental ill health among mothers [45, 46]. Social support can be theorized to be either a mediator or effect modifier; since building social support is a key feature of the integrated intervention is the building of supportive relationships, we hypothesize that increased social support will mediate the relationship between the intervention and child outcomes. *Maternal distress and empowerment*: Maternal distress will be assessed using SRQ and maternal empowerment will be assessed using two items previously used in the same population [32]. *Breastfeeding and other health related variables*; Perinatal depression interventions similar to integrated intervention have resulted in higher rates of exclusive breastfeeding at 6 months and increased likelihood of completion of scheduled immunizations [6]. These health-related behaviours may contribute to the maternal wellbeing intervention's effect on the infant and so we will assess behaviours related to feeding (breastfeeding, introduction of solid food) and immunizations.

Cost-effectiveness analysis

Health economic analysis will be conducted to determine the difference in costs and outcomes in the intervention as compared to the control arm. The cost of the intervention will be calculated based on the sum of materials, personnel training, and delivery. We will then compare the combination of the cost of the intervention and the service cost data with mother-child interaction summary scores. If the intervention results in more favourable summary scores (improvement in mother-infant interaction) and lower total costs, it will be described as superior to no intervention option. If, in contrast, the intervention results in more favourable scores but is also more expensive, then the cost-effectiveness calculation will be based on the cost and scores of mother-infant interaction outcomes.

Primary analysis will be of total costs over the 12-months post-partum follow-up treatment period. Recognizing that cost data are often skewed, the bootstrap technique will be applied. The sampling with replacement from original observed pairs of costs and effects will be employed to maintain correlation structure between costs and benefits, and bootstrapping sampling will be repeated 1000 times. For each bootstrap sample, an estimate of differential total mean costs and expected mean effectiveness will be calculated. The 95% CIs for the differential estimates will be derived from the 25th and 97.5th percentiles [47, 48].

Cost-effectiveness will be assessed by combing costs with the primary outcome measure (i.e. mother-infant interaction) scores in incremental cost-effectiveness analysis. Repeat resampling from the costs and effectiveness data (bootstrapping) will be used to calculate the probability that each of the treatment is the optimal choice, subject to a range of possible maximum values (ceiling ratio) that a decision-maker might be willing to pay for a unit improvement in mother-infant interaction scores. The results of the cost-effectiveness will be reported as incremental cost-

For peer review only - http://bmjopen.bmj.com/site/about/guidelines.xhtml

effectiveness ratios, and acceptability curves which summarise the information contained in a cost-effectiveness plane [49].

Mixed-methods evaluation

We will evaluate the feasibility of our intervention program using mixed methods study design. Mixed-methods evaluation of implementation will be carried out to assess assumptions underlying the intervention strategy and will cover other aspects of program implementation such as number of research participants who signed-up and complete the intervention and satisfaction with intervention, difficulties and successes in carrying out intervention activities, acceptability, feasibility and appropriateness of using integrated intervention.

Satisfaction with the intervention and barriers and facilitators to implementation, feasibility of workload, and difficulties and successes in carrying out intervention activities and satisfaction with training and supervision will be explored through semi-structured interviews with the relevant stakeholders.

Trial management

Trial management will be provided by Trial Steering Committee (TSC) that comprises Principal Investigator (PI), Co-Investigator (s), trial coordinator, senior researchers and intervention staff who will meet monthly.

Dissemination

There is a growing evidence base on the feasibility, acceptability and effectiveness of peer/parent delivered, psychosocial and early child development interventions [6, 16, 50]. Training and supervision of large numbers of health workers in new interventions is a key barrier to scale up of programs. Our intervention combines evidence-based elements into a single package, making delivery more effective and synergistic, through a single delivery agent i.e. non-specialist facilitator. The use of non-specialist facilitators in disseminating the interventions has the potential to extend the reach of the intervention in a cost-effective fashion. If proven effective, the study will contribute to scale-up care for maternal and child mental health in low resource settings globally. We will disseminate our results to key stakeholders. Dissemination activities will include publication of study results in open access, peer-reviewed journals, and presentations at academic conferences and community forums.

References:

1. Black, R., et al., *Maternal and child undernutrition and overweight in low-income and middle-income countries.* The lancet, 2013. **382**(9890): p. 427-451.
2. Grantham-McGregor, S., et al., *Developmental potential in the first 5 years for children in developing countries.* The lancet, 2007. **369**(9555): p. 60-70.
3. Fisher, J., et al., *Prevalence, nature, severity and correlates of postpartum depressive symptoms in Vietnam.* BJOG: An International Journal of Obstetrics & Gynaecology, 2004. **111**(12): p. 1353-1360.
4. Rahman, A., et al., *Impact of maternal depression on infant nutritional status and illness: a cohort study.* Archives of general psychiatry, 2004. **61**(9): p. 946-952.
5. Hurley, K.M., A.K. Yousafzai, and F. Lopez-Boo, *Early child development and nutrition: a review of the benefits and challenges of implementing integrated interventions.* J Advances in nutrition, 2016. **7**(2): p. 357-363.
6. Rahman, A., et al., *Cognitive behaviour therapy-based intervention by community health workers for mothers with depression and their infants in rural Pakistan: a cluster-randomised controlled trial.* The Lancet, 2008. **372**(9642): p. 902-909.

For peer review only - http://bmjopen.bmj.com/site/about/guidelines.xhtml

7. Yousafzai, A.K., et al., *Effect of integrated responsive stimulation and nutrition interventions in the Lady Health Worker programme in Pakistan on child development, growth, and health outcomes: a cluster-randomised factorial effectiveness trial.* The Lancet, 2014. **384**(9950): p. 1282-1293.

8. Yousafzai, A.K., et al., *Effects of responsive stimulation and nutrition interventions on children's development and growth at age 4 years in a disadvantaged population in Pakistan: a longitudinal follow-up of a cluster-randomised factorial effectiveness trial.* The Lancet Global Health, 2016. **4**(8): p. e548-e558.

9. Stevens, G.A., et al., *Trends in mild, moderate, and severe stunting and underweight, and progress towards MDG 1 in 141 developing countries: a systematic analysis of population representative data.* 2012. **380**(9844): p. 824-834.

10. Rahman, R. Harrington, and J. Bunn, *Can maternal depression increase infant risk of illness and growth impairment in developing countries?* Journal of Child Care, health and development 2002. **28**(1): p. 51-56.

11. Hamdani, S.U., et al., *Family networks to improve outcomes in children with intellectual and developmental disorders: a qualitative study.* J International journal of mental health systems, 2014. **8**(1): p. 7.

12. Chorpita, B.F., E.L. Daleiden, and J.R. Weisz, *Identifying and selecting the common elements of evidence based interventions: A distillation and matching model.* Mental health services research, 2005. **7**(1): p. 5-20.

13. UNICEF. *Care for Child Development Package*. 2012; Available from: https://www.unicef.org/earlychildhood/index_68195.html.

14. Salomone, E., et al., *Development of the WHO Caregiver Skills Training Program for Developmental Disorders or Delays.* 2019. **10**(769).

15. WHO. *Thinking Healthy*. 2015 [cited 2020 Sep 16]; Available from: https://www.who.int/mental_health/maternal-child/thinking_healthy/en/.

16. Zafar, S., et al., *Integrating maternal psychosocial well-being into a child-development intervention: the five-pillars approach.* Ann N Y Acad Sci, 2014. **1308**: p. 107-17.

17. NIPS. *Pakistan demographic and health survey 2012–13.* 2013 [cited 2015 April 15]; Available from: https://dhsprogram.com/pubs/pdf/fr290/fr290.pdf.

18. Rahman, A. and F. Creed, *Outcome of prenatal depression and risk factors associated with persistence in the first postnatal year: Prospective study from Rawalpindi, Pakistan.* Journal of Affective Disorders, 2007. **100**(1-3): p. 115-121.

19. Bureau of Statistics and UNICEF Pakistan, *District based Multiple Indicator Cluster Survey (MICS), 2003-2004: Punjab*. 2003, Government of Punjab, Planning and Development Department, Federal Bureau of Statistics Islamabad United Nations Children's Fund, 2004: Lahore

20. Rasheed, M.A. and A.K. Yousafzai, *The development and reliability of an observational tool for assessing mother–child interactions in field studies‐experience from Pakistan.* Child: care, health and development, 2015. **41**(6): p. 1161-1171.

21. Landry, S.H., et al., *Does early responsive parenting have a special importance for children's development or is consistency across early childhood necessary?* Developmental psychology, 2001. **37**(3): p. 387.

22. Albers, C.A. and A.J. Grieve, *Test review: Bayley, N.(2006). Bayley scales of infant and toddler development–third edition. San Antonio, TX: Harcourt assessment.* Journal of Psychoeducational Assessment, 2007. **25**(2): p. 180-190.

23. Squires, J., D.D. Bricker, and E. Twombly, *Ages & stages questionnaires*. 2009: Paul H. Brookes Baltimore, MD.

24. Caldwell, B. and R. Bradley, *Home observation for measurement of the environment*. 1984: University of Arkansas at Little Rock Little Rock.

25. WHO Expert Committee, *Physical status: the use and interpretation of anthropometry.* http://helid. digicollection. Org/en/d/Jh0211e/, 1995.

For peer review only - http://bmjopen.bmj.com/site/about/guidelines.xhtml

26. Sikander, S., et al., *Cognitive-behavioral counseling for exclusive breastfeeding in rural pediatrics: a cluster RCT.* Pediatrics, 2015: p. peds. 2014-1628.
27. Rahman, A., et al., *Maternal depression increases infant risk of diarrhoeal illness:–a cohort study.* Archives of Disease in Childhood, 2007. **92**(1): p. 24-28.
28. Beusenberg, M., J.H. Orley, and W.H. Organization, *A User's guide to the self reporting questionnaire (SRQ*. 1994, Geneva: World Health Organization.
29. Varni, J.W., M. Seid, and C.A. Rode, *The PedsQL™: measurement model for the pediatric quality of life inventory.* Medical care, 1999: p. 126-139.
30. Dahlem, N.W., G.D. Zimet, and R.R. Walker, *THE MULTIDIMENSIONAL SCALE OF PERCEIVED SOCIAL SUPPORT - A CONFIRMATION STUDY.* Journal of Clinical Psychology, 1991. **47**(6): p. 756-761.
31. Üstün, T.B., et al., *Developing the World Health Organization disability assessment schedule 2.0.* Bulletin of the World Health Organization, 2010. **88**: p. 815-823.
32. Rahman, A., et al., *Effective treatment of perinatal depression for women in debt and lacking financial empowerment in a low-income country.* The British Journal of Psychiatry, 2012. **201**(6): p. 451-457.
33. Schuler, S.R., S.M. Hashemi, and A.P. Riley, *The influence of women's changing roles and status in Bangladesh's fertility transition: evidence from a study of credit programs and contraceptive use.* World Development, 1997. **25**(4): p. 563-575.
34. Moher, D., et al., *CONSORT 2010 Explanation and Elaboration: updated guidelines for reporting parallel group randomised trials.* 2010. **340**: p. c869.
35. Enders, C.K., *Applied missing data analysis*. 2010: Guilford press.
36. Appelbaum, M., et al., *The effects of infant child care on infant-mother attachment security: Results of the NICHD study of early child care.* Child Development, 1997. **68**(5): p. 860-879.
37. Black, M., et al., *Depressive symptoms among rural Bangladeshi mothers: implications for infant development.* Journal of Child Psychology and Psychiatry, 2007. **48**(8): p. 764-772.
38. Schmid, B., et al., *Quality of early mother-child interaction associated with depressive psychopathology in the offspring: A prospective study from infancy to adulthood.* Journal of Psychiatric Research, 2011. **45**(10): p. 1387-1394.
39. Tamis-LeMonda, C.S., M.H. Bornstein, and L. Baumwell, *Maternal responsiveness and children's achievement of language milestones.* Child Development, 2001. **72**(3): p. 748-767.
40. van Doesum, K.T.M., et al., *A randomized controlled trial of a home-visiting intervention aimed at preventing relationship problems in depressed mothers and their infants.* Child Development, 2008. **79**(3): p. 547-561.
41. Caldwell, B. and R. Bradley, *Home observation for measurement of the environment: Administration manual*. 2003, Family & Human Dynamics Research Institute, Arizone State University: Tempe, AZ.
42. Aboud, F.E., *Evaluation of an early childhood parenting programme in rural Bangladesh.* Journal of Health Population and Nutrition, 2007. **25**(1): p. 3-13.
43. Aboud, F.E. and S. Akhter, *A Cluster-Randomized Evaluation of a Responsive Stimulation and Feeding Intervention in Bangladesh.* Pediatrics, 2011. **127**(5): p. E1191-E1197.
44. Beeber, L.S., et al., *Short-Term In-Home Intervention Reduces Depressive Symptoms in Early Head Start Latina Mothers of Infants and Toddlers.* Research in Nursing & Health, 2010. **33**(1): p. 60-76.
45. Fisher, J., et al., *Prevalence and determinants of common perinatal mental disorders in women in low- and lower-middle-income countries: a systematic review.* Bulletin of the World Health Organization, 2012. **90**(2): p. 139-149.
46. Rahman, A., Z. Iqbal, and R. Harrington, *Life events, social support and depression in childbirth: perspectives from a rural community in the developing world.* Psychological Medicine, 2003. **33**(7): p. 1161-1167.

For peer review only

For peer review only - http://bmjopen.bmj.com/site/about/guidelines.xhtml

47. Barber, J.A. and S.G. Thompson, *Analysis and interpretation of cost data in randomised controlled trials: review of published studies.* BMJ, 1998. **317**(7167): p. 1195-1200.
48. Efron, B. and R. Tibshirani, *An Introduction to the Bootstrap, vol. 23, no. 2*. 1998, Chapman & Hall, New York.
49. Fenwick, E., K. Claxton, and M. Sculpher, *Representing uncertainty: the role of cost-effectiveness acceptability curves.* Health economics, 2001. **10**(8): p. 779-787.
50. Hamdani, S.U., et al., *WHO Parents Skills Training (PST) programme for children with developmental disorders and delays delivered by Family Volunteers in rural Pakistan: study protocol for effectiveness implementation hybrid cluster randomized controlled trial.* Global mental health, 2017. **4**.

Acknowledgment

The study sponsor and funders have no role in the study design; collection, management, analysis, and interpretation of data; writing of the report; and the decision to submit the report for publication.

Authors' Contributors

SUH, ZeH & DW will have the access of all datasets. ZeH drafted the initial manuscript. SUH conceived the study and wrote the final manuscript. AG, FS, AR, BM, DW & AR contributed to the writing. All authors approved of the final version of the manuscript.

Funding statement

This work is supported by Grand Challenges Canada, Saving Brains, Grant Number SB-POC-1809-18680

Competing interests statement None declared.

Word count 4515 (without Tables and Figure)

For peer review only - http://bmjopen.bmj.com/site/about/guidelines.xhtml

Figure 1: Flow Diagram

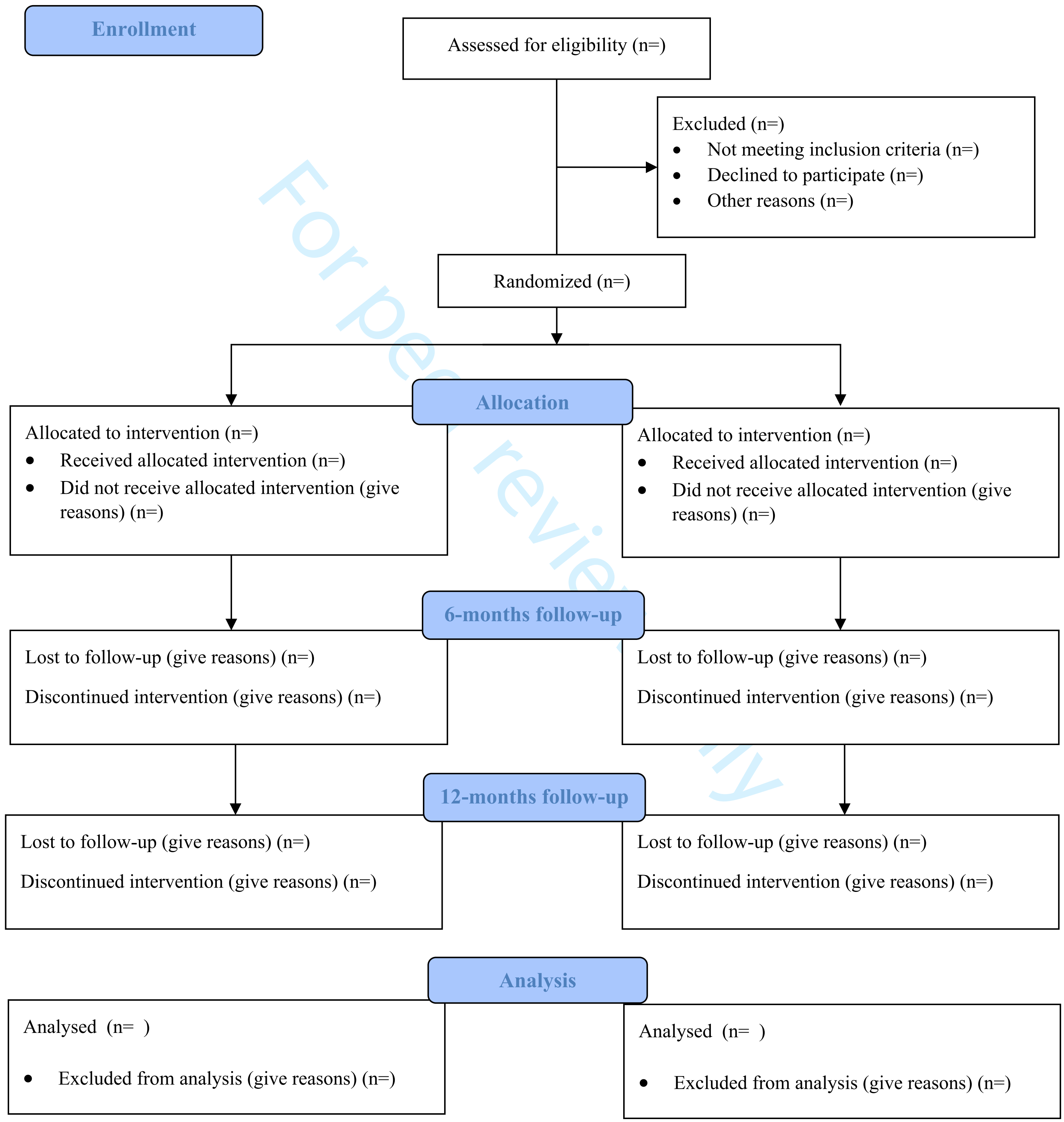

For peer review only

STANDARD PROTOCOL ITEMS: RECOMMENDATIONS FOR INTERVENTIONAL TRIALS

SPIRIT 2013 Checklist: Recommended items to address in a clinical trial protocol and related documents*

Section/item	Item No	Description	Addressed on page no.
**Administrative information**			
Title	1	Descriptive title identifying the study design, population, interventions, and, if applicable, trial acronym	Title p.1
Trial registration	2a	Trial identifier and registry name. If not yet registered, name of intended registry	2
	2b	All items from the World Health Organization Trial Registration Data Set	Included in the registration
Protocol version	3	Date and version identifier	N/A
Funding	4	Sources and types of financial, material, and other support	18
Roles and responsibilities	5a	Names, affiliations, and roles of protocol contributors	1 & 18
	5b	Name and contact information for the trial sponsor	N/A
	5c	Role of study sponsor and funders, if any, in study design; collection, management, analysis, and interpretation of data; writing of the report; and the decision to submit the report for publication, including whether they will have ultimate authority over any of these activities	18
	5d	Composition, roles, and responsibilities of the coordinating centre, steering committee, endpoint adjudication committee, data management team, and other individuals or groups overseeing the trial, if applicable (see Item 21a for data monitoring committee)	15
**Introduction**			
Background and rationale	6a	Description of research question and justification for undertaking the trial, including summary of relevant studies (published and unpublished) examining benefits and harms for each intervention	3

For peer review only

	6b	Explanation for choice of comparators	9
Objectives	7	Specific objectives or hypotheses	3
Trial design	8	Description of trial design including type of trial (eg, parallel group, crossover, factorial, single group), allocation ratio, and framework (eg, superiority, equivalence, noninferiority, exploratory)	3
**Methods: Participants, interventions, and outcomes**			
Study setting	9	Description of study settings (eg, community clinic, academic hospital) and list of countries where data will be collected. Reference to where list of study sites can be obtained	4
Eligibility criteria	10	Inclusion and exclusion criteria for participants. If applicable, eligibility criteria for study centres and individuals who will perform the interventions (eg, surgeons, psychotherapists)	4
Interventions	11a	Interventions for each group with sufficient detail to allow replication, including how and when they will be administered	5-9
	11b	Criteria for discontinuing or modifying allocated interventions for a given trial participant (eg, drug dose change in response to harms, participant request, or improving/worsening disease)	N/A
	11c	Strategies to improve adherence to intervention protocols, and any procedures for monitoring adherence (eg, drug tablet return, laboratory tests)	N/A
	11d	Relevant concomitant care and interventions that are permitted or prohibited during the trial	9
Outcomes	12	Primary, secondary, and other outcomes, including the specific measurement variable (eg, systolic blood pressure), analysis metric (eg, change from baseline, final value, time to event), method of aggregation (eg, median, proportion), and time point for each outcome. Explanation of the clinical relevance of chosen efficacy and harm outcomes is strongly recommended	10-12
Participant timeline	13	Time schedule of enrolment, interventions (including any run-ins and washouts), assessments, and visits for participants. A schematic diagram is highly recommended	12 & 19

For peer review only

		(see Figure)	
Sample size	14	Estimated number of participants needed to achieve study objectives and how it was determined, including clinical and statistical assumptions supporting any sample size calculations	4 & 5
Recruitment	15	Strategies for achieving adequate participant enrolment to reach target sample size	N/A
**Methods: Assignment of interventions (for controlled trials)**			
Allocation:			
Sequence generation	16a	Method of generating the allocation sequence (eg, computer-generated random numbers), and list of any factors for stratification. To reduce predictability of a random sequence, details of any planned restriction (eg, blocking) should be provided in a separate document that is unavailable to those who enrol participants or assign interventions	12
Allocation concealment mechanism	16b	Mechanism of implementing the allocation sequence (eg, central telephone; sequentially numbered, opaque, sealed envelopes), describing any steps to conceal the sequence until interventions are assigned	12
Implementation	16c	Who will generate the allocation sequence, who will enrol participants, and who will assign participants to interventions	12
Blinding (masking)	17a	Who will be blinded after assignment to interventions (eg, trial participants, care providers, outcome assessors, data analysts), and how	12
	17b	If blinded, circumstances under which unblinding is permissible, and procedure for revealing a participant's allocated intervention during the trial	12
**Methods: Data collection, management, and analysis**			
Data collection methods	18a	Plans for assessment and collection of outcome, baseline, and other trial data, including any related processes to promote data quality (eg, duplicate measurements, training of assessors) and a description of study instruments (eg, questionnaires, laboratory tests) along with their reliability and validity, if known. Reference to where data collection forms can be found, if not in the protocol	10-12
	18b	Plans to promote participant retention and complete follow-up, including list of any outcome data to be collected for participants who discontinue or deviate from	N/A

For peer review only

For peer review only - http://bmjopen.bmj.com/site/about/guidelines.xhtml

		intervention protocols	
Data management	19	Plans for data entry, coding, security, and storage, including any related processes to promote data quality (eg, double data entry; range checks for data values). Reference to where details of data management procedures can be found, if not in the protocol	13
Statistical methods	20a	Statistical methods for analysing primary and secondary outcomes. Reference to where other details of the statistical analysis plan can be found, if not in the protocol	13
	20b	Methods for any additional analyses (eg, subgroup and adjusted analyses)	14-15
	20c	Definition of analysis population relating to protocol non-adherence (eg, as randomised analysis), and any statistical methods to handle missing data (eg, multiple imputation)	13
**Methods: Monitoring**			
Data monitoring	21a	Composition of data monitoring committee (DMC); summary of its role and reporting structure; statement of whether it is independent from the sponsor and competing interests; and reference to where further details about its charter can be found, if not in the protocol. Alternatively, an explanation of why a DMC is not needed	15
	21b	Description of any interim analyses and stopping guidelines, including who will have access to these interim results and make the final decision to terminate the trial	N/A
Harms	22	Plans for collecting, assessing, reporting, and managing solicited and spontaneously reported adverse events and other unintended effects of trial interventions or trial conduct	12-13
Auditing	23	Frequency and procedures for auditing trial conduct, if any, and whether the process will be independent from investigators and the sponsor	N/A
**Ethics and dissemination**			
Research ethics approval	24	Plans for seeking research ethics committee/institutional review board (REC/IRB) approval	12
Protocol amendments	25	Plans for communicating important protocol modifications (eg, changes to eligibility criteria, outcomes, analyses) to relevant parties (eg, investigators, REC/IRBs, trial participants, trial registries, journals, regulators)	N/A
Consent or assent	26a	Who will obtain informed consent or assent from potential trial participants or authorised surrogates, and how (see Item 32)	5

For peer review only

	26b	Additional consent provisions for collection and use of participant data and biological specimens in ancillary studies, if applicable	N/A
Confidentiality	27	How personal information about potential and enrolled participants will be collected, shared, and maintained in order to protect confidentiality before, during, and after the trial	13
Declaration of interests	28	Financial and other competing interests for principal investigators for the overall trial and each study site	18
Access to data	29	Statement of who will have access to the final trial dataset, and disclosure of contractual agreements that limit such access for investigators	18
Ancillary and post-trial care	30	Provisions, if any, for ancillary and post-trial care, and for compensation to those who suffer harm from trial participation	N/A
Dissemination policy	31a	Plans for investigators and sponsor to communicate trial results to participants, healthcare professionals, the public, and other relevant groups (eg, via publication, reporting in results databases, or other data sharing arrangements), including any publication restrictions	15
	31b	Authorship eligibility guidelines and any intended use of professional writers	N/A
	31c	Plans, if any, for granting public access to the full protocol, participant-level dataset, and statistical code	N/A
**Appendices**			
Informed consent materials	32	Model consent form and other related documentation given to participants and authorised surrogates	N/A
Biological specimens	33	Plans for collection, laboratory evaluation, and storage of biological specimens for genetic or molecular analysis in the current trial and for future use in ancillary studies, if applicable	N/A

*It is strongly recommended that this checklist be read in conjunction with the SPIRIT 2013 Explanation & Elaboration for important clarification on the items. Amendments to the protocol should be tracked and dated. The SPIRIT checklist is copyrighted by the SPIRIT Group under the Creative Commons "Attribution-NonCommercial-NoDerivs 3.0 Unported" license.

For peer review only

For peer review only - http://bmjopen.bmj.com/site/about/guidelines.xhtml

BMJ Open

Evaluating impact of a common elements based intervention to improve maternal psychological wellbeing and mother-infant interaction in rural Pakistan: Study protocol for a randomized controlled trial

Journal:	*BMJ Open*
Manuscript ID	bmjopen-2020-047609.R1
Article Type:	Protocol
Date Submitted by the Author:	14-May-2021
Complete List of Authors:	Huma, Zill-e-; Human Development Research Foundation, Implementation Science; University of Liverpool Faculty of Health and Life Sciences, Department of Primary Care and Mental Health<br>Gillani, Ayella; Human Development Research Foundation, Implementation Science<br>Shafique, Fakhira; Human Development Research Foundation, Implementation Science<br>Rashid, Alina; Human Development Research Foundation, Implementation Science<br>Mahjabeen, Bushra; Human Development Research Foundation, Implementation Science<br>Javed, Hashim; Human Development Research Foundation, Implementation Science<br>Wang, Duolao; Liverpool School of Tropical Medicine, Department of Clinical Sciences<br>Rahman, Atif; University of Liverpool Faculty of Health and Life Sciences, Department of Primary Care and Mental Health<br>Hamdani, Syed Usman; University of Liverpool Faculty of Health and Life Sciences, Department of Primary Care and Mental Health ; Human Development Research Foundation, Implementation Science
**Primary Subject Heading**:	Mental health
Secondary Subject Heading:	Mental health, Evidence based practice
Keywords:	MENTAL HEALTH, Community child health < PAEDIATRICS, Depression & mood disorders < PSYCHIATRY, Public health < INFECTIOUS DISEASES, Developmental neurology & neurodisability < PAEDIATRICS, Anxiety disorders < PSYCHIATRY

SCHOLARONE™
Manuscripts

I, the Submitting Author has the right to grant and does grant on behalf of all authors of the Work (as defined in the below author licence), an exclusive licence and/or a non-exclusive licence for contributions from authors who are: i) UK Crown employees; ii) where BMJ has agreed a CC-BY licence shall apply, and/or iii) in accordance with the terms applicable for US Federal Government officers or employees acting as part of their official duties; on a worldwide, perpetual, irrevocable, royalty-free basis to BMJ Publishing Group Ltd ("BMJ") its licensees and where the relevant Journal is co-owned by BMJ to the co-owners of the Journal, to publish the Work in this journal and any other BMJ products and to exploit all rights, as set out in our licence.

The Submitting Author accepts and understands that any supply made under these terms is made by BMJ to the Submitting Author unless you are acting as an employee on behalf of your employer or a postgraduate student of an affiliated institution which is paying any applicable article publishing charge ("APC") for Open Access articles. Where the Submitting Author wishes to make the Work available on an Open Access basis (and intends to pay the relevant APC), the terms of reuse of such Open Access shall be governed by a Creative Commons licence – details of these licences and which Creative Commons licence will apply to this Work are set out in our licence referred to above.

Other than as permitted in any relevant BMJ Author's Self Archiving Policies, I confirm this Work has not been accepted for publication elsewhere, is not being considered for publication elsewhere and does not duplicate material already published. I confirm all authors consent to publication of this Work and authorise the granting of this licence.

For peer review only

Evaluating impact of a common elements based intervention to improve maternal psychological wellbeing and mother-infant interaction in rural Pakistan: Study protocol for a randomized controlled trial

Zill-e-Huma [1, 2], Ayella Gillani [1], Fakhira Shafique [1], Alina Rashid [1], Bushra Mahjabeen [1] Hashim Javed[1], Duolao Wang [3], Atif Rahman [2], Syed Usman Hamdani [1, 2]

[1]Human Development Research Foundation, Rawalpindi, Pakistan

[2]Department of Primary Care and Mental Health, University of Liverpool, Liverpool, United Kingdom

[3]Liverpool School of Tropical Medicine, Liverpool, United Kingdom

Corresponding Author:
Zill-e-Huma
Human Development Research Foundation (HDRF), Pakistan
E: zille.huma@hdrfoundation.org

For peer review only - http://bmjopen.bmj.com/site/about/guidelines.xhtml

Abstract

Introduction Millions of children in low resource settings are at high risk of poor development due to factors such as under nutrition, inadequate stimulation and maternal depression. Evidence-based interventions to address these risk factors exist, but often as a separate and overlapping package. The current study aims to evaluate the effectiveness of a common elements-based intervention to improve mother-infant interaction at 12-months post-partum.

Method and analysis A two-arm, single-blinded, individual randomized controlled trial (RCT) is being carried out in the community settings of the rural sub-district of Gujar Khan in Rawalpindi, Pakistan. 250 pregnant women in third trimester with distress (Self-Reporting Questionnaire (SRQ), cut-off score >9) have been randomized on 1:1 allocation ratio into intervention (n=125) and Treatment as Usual (TAU) arms (n=125). The participants in the intervention arm will receive 15 individual sessions of intervention on a monthly basis by non-specialist facilitators. The intervention involves components of early stimulation, learning through play, responsive feeding, guided discovery using pictures, behavioural activation and problem solving. The primary outcome is caregiver-infant interaction at 12-months post-partum. The secondary outcomes include maternal psychological wellbeing, quality of life, social support and empowerment. Infant secondary outcomes include growth, nutrition and development. The data will be collected at baseline, 6- and 12-months' post-partum. A qualitative process evaluation will be conducted to inform the feasibility of intervention delivery.

Trial registration: Registered prospectively with ClinicalTrials.gov as "a common elements-based intervention to improve maternal psychological well-being and mother-infant interaction". Identifier: NCT04252807. Study start and end date: September 2019 to March, 2021 (19-months).

Keywords: Maternal distress; mother-infant interaction; common elements treatment approach; nurturing care; low resource settings

Article summary

- To address the burden associated with poor infant development and perinatal distress we have developed a common elements based early intervention. The intervention is designed to be delivered through non-specialist facilitators in low resource community settings.
- In a single-blinded, individual randomized controlled trial, we will evaluate the impact of intervention using a pragmatic primary outcome measure i.e. 5-minute observation of a live caregiver-child interaction.
- The COVID-19 pandemic has resulted in limited face to face contact with the study participants and the intervention is being delivered via telephone or in-person sessions where possible. We shall evaluate the feasibility and acceptability of blended intervention delivery methodology using mixed methods process evaluation.
- Continued participation of mother-infant dyads through the completion of the intervention and to the final assessment point at 12-months postpartum could be a challenge in the COVID-19 pandemic.

For peer review only - http://bmjopen.bmj.com/site/about/guidelines.xhtml

Introduction

In South Asia, 35-45% children are undernourished [1] and over 40 million lag behind in their cognitive and socio-emotional development [2]. The situation is compounded by maternal depression, which affects over a quarter of all women [3]. Suboptimal functioning and agency in depressed mothers is a barrier to implementation of any intervention directed at the young child [4]. Together, these factors have a negative impact on provision of nurturing care that is essential for optimal development [5-8].

Pakistan has a population of about 200 million people, of which three quarters live in rural areas. 47% of children under 5 years of age suffer from stunted growth and development in Pakistan [9]. By conservative estimate about eight million children do not achieve their full cognitive potential [2]. Prevalence studies in the proposed study area have reported high rates of perinatal depression in mothers ranging from 25-37% and a strong and independent association of maternal depression with low birth weight and under nutrition [10]. Studies have shown that antenatal stress can have detrimental effects on fetal development [11]. Lack of early interventions mean that many of these children might develop cognitive, social and emotional difficulties, contributing further to emotional and financial stress in the families [12]. Especially in the context of on-going COVID-19 pandemic, which is indirectly impacting maternal and child health outcomes due to disruption of health care services and lack of access to existing maternal and child health care during lockdown, delivering early interventions in community settings becomes imperative [13].

Considerable effort has gone into child survival programmes, leading to a reduction in child mortality rates over the last decade. However, improved child survival means there are many more children who are at-risk of developmental delay. Evidence-based interventions to address risk factors for poor child development exist, but often as separate and overlapping packages of interventions.

To address these challenges, we have developed a package of intervention using a common elements approach [14] that combines evidence-based elements from packages of care addressing early infant stimulation, responsive feeding and maternal distress [15-17]. These approaches recognize the importance of early critical years and role of the primary caregiver – normally the mother to provide nutrition and an optimal environment for interaction and stimulation. CEA is the concept of identifying components that overlap across multiple evidence-based practices for a specific problem area [14]. CEA combines a number of overlapping theoretical approaches into a single intervention with synergistic effects across the range of domains. The mother, in turn, receives psychosocial support, education and skills training specific to Early Child Development (ECD), provided via a well monitored and supervised delivery system.

We aim to evaluate the effectiveness of common elements-based intervention compared to usual care to improve mother-infant interaction along with improvements in maternal psychological wellbeing, infant growth, nutrition and development at 12-months post-partum in low resource rural community settings of Pakistan.

Hypotheses

We hypothesize that common element based integrated intervention will result in improving caregiver-child interaction measured using Observation of Mother and Child Interaction (OMCI) in intervention arm compared with the Treatment –As-Usual in control arm, at 12 months' postpartum. Our secondary hypotheses (exploratory) are that our intervention will result in improvement in infant growth, nutrition and development and will improve maternal psychological wellbeing and quality of life.

Methods and analysis

For peer review only - http://bmjopen.bmj.com/site/about/guidelines.xhtml

Study design

A two-arm, single-blinded, individual randomized controlled trial with mixed-methods process monitoring and evaluation is being carried out to evaluate the effectiveness of intervention. The participants are recruited from a rural sub-district, randomized into intervention and control arms, on a 1:1 allocation ratio.

Patient and public involvement

The multicomponent intervention is based on existing community programmes developed by the research team working collaboratively with the people from local communities including mothers with post-natal depression, fathers, extended family members, community health workers and local volunteers [4, 6, 12, 18]. The relevant stakeholders (community representatives and gate keepers and mental health experts) were involved through consultative workshops (Theory of Change workshops) in the formative research to develop a hypothesized pathway for the implementation of intervention in the study sub-district. The findings of the present study will be disseminated to participants through presentation at community forums.

Study settings

The present study is being conducted in sub-district of Gujar Khan in Rawalpindi Pakistan. The sub-district represents a typical rural area of Pakistan and have poor demographic and health profile indicators (with high fertility rate – 4 births per woman and low female literacy rate i.e. 45%) [19], where 1 in every 4 women suffer from perinatal depression [19, 20]. The infant mortality rate in Rawalpindi has been estimated to be 55 per thousand live births, the under-5 mortality rate is 82 per thousand live births and the percentage of underweight children below 5 years of age is 25% [21]. Primary health care is delivered through a network of Basic Health Units (BHUs), each providing care to about 15,000–20,000 people. Each BHU is staffed by a doctor, midwife, vaccinator, and 15–20 village-based Community Health Workers (CHWs) also called Lady Health Workers (LHWs). These women have completed secondary school and are trained to provide preventive maternal and child health care, and education in the community. Each LHW is responsible for about 100 households in her village. The participants shall be recruited from the catchment areas of LHWs. The study participants are permanent residents in the study sub-district and have been living here for past many generations. The implementing research organization has been working closely with the health department in the study area for last 15 years and has developed a trustable relationship with the community members. The study participants are being approached through Lady Health Workers (LHWs), who have trusting relationship with community, this makes the enrolment process and retention in the study more feasible. Also, convenience of participants is taken into account when assessment and intervention delivery sessions are scheduled. These factors are expected to improve the retention rate of participants in study over a longer period of time.

Research participants:

The target population for the present study is pregnant women in third trimester with distress (screen positive on Self-Reporting Questionnaire, cut-off score ≥ 9).

Eligibility criteria of participants

Inclusion criteria:

- Pregnant with third trimester (28 gestational week)
- Age 18-40 years
- Intend to reside in the study areas until the completion of the study

For peer review only - http://bmjopen.bmj.com/site/about/guidelines.xhtml

- Score ≥9 on the SRQ

Exclusion criteria

- Women who require immediate or on-going medical or psychiatric care reported.
- Severe previous or current obstetric morbidity including eclampsia and antepartum hemorrhage;
- Medical disorders that require inpatient management (e.g., diabetes, hypertension, thromboembolism, cardiac disease)

Sample size calculations

The power calculations are based on one primary outcome, i.e. mother-child interaction on Observation of Mother and Child Interaction (OMCI) tool [22] at 12 months' post-partum. The scoring of tool is based on the frequency of the occurrence of responsive behaviours of mother with higher scores indicating more responsive interactions. The effect size for the present study was estimated based on the findings of a similar study conducted in Pakistan, where OMCI was used as one of the outcomes to evaluate the effect of responsive stimulation intervention on caregiver-child interaction at 12 and 24-months post-partum [8]. The findings of the study showed mother–child dyads receiving responsive caregiving intervention had significantly higher mean scores on OMCI at 12 months (mean 32.3 [SD 8.3] vs mean 27.1 [SD 8.2], p <0.0001) than those who were allocated to control arm. Moreover, repeated-measures analysis showed that effects were sustained at 24-months. As the effect sizes for OMCI in Yousafzai et al 2015's study ranged between 0.6 to 0.9; therefore, a conservative effect size of 0.4 (two-sides hypothesis) at 12-months post-partum was proposed in the present study. The effect size was calculated using the two-sided t-test. Assuming an effect size of 0.4, with 80% power, 0.05 significance, and a two-sided hypothesis test and accounting for 20% attrition, we will need 250 caregiver-child dyads (125 in each arm).

Recruitment procedure

Before recruiting participants in the trial, the local community stakeholders were taken on-board. As mentioned in the study settings, the current study is being conducted in the rural sub-district of Gujar Khan, Rawalpindi district, Pakistan. The study sub-district consists of 36 Union Councils (UCs)- the smallest administrative units of the sub-district. Each UC has a Basic Health Unit (BHU) that provides primary health care to about 15,000–20,000 local population. Each BHU is staffed by a doctor, midwife, vaccinator, and 15–20 village-based Community Health Workers (CHWs) also called Lady Health Workers (LHWs). These women have completed secondary school and are trained to provide preventive maternal and child health care, and education in the community. Each LHW is responsible for about 100-150 households in her village and keeps a register of every new pregnancy in her catchment area. The research participants in the present study is being recruited from the catchment areas of LHWs, where, every woman in their third trimester of pregnancy is being assessed for eligibility. Pregnant women in their third trimester (N=250), who fulfil the eligibility criteria, are being enrolled in the trial and individualized randomized into intervention and TAU arm (1:1 ratio). (Fig 1). Written informed consent is obtained from the study participants by trained assessment team. A complete record of assessment against eligibility criteria, and reasons for refusal is maintained for those who do not consent for participation in the trial.

All potential eligible pregnant women in third trimester are screened for psychological distress by research team using Self-Reporting Questionnaire (SRQ). Psychological distress is represented by

For peer review only - http://bmjopen.bmj.com/site/about/guidelines.xhtml

subscales of depression/anxiety, somatic symptoms, reduced vital energy, and depressive thoughts during the past month. The scale reflects multidimensional nature of mental illnesses. It has 20 items scored on 0 or 1. A score of 1 indicates that the symptom was present during the past month; a score of 0 indicates that the symptom was absent. The maximum score is therefore 20, with a higher score indicating higher psychological distress. A score of ≥ 9 indicates increased risk of postnatal depression [8] and mothers with these scores have been recruited in the study.

Intervention

The intervention called 'Nurturing Care' combines common elements from evidence-based interventions for maternal psychosocial distress (e.g. WHO Thinking Healthy Program [17]), early stimulation and responsive feeding (e.g. UNICEF Care for Child Development Package [15]), and child care (e.g. WHO Caregivers Skills Training program [16]) for optimal development of at risk mother and children.

The intervention package aims to improve a) mother psychological distress by promoting problem solving, developing empathic listening, improving family support, using guided discovery through pictures and behavioural activation; b) child development through psycho-education, responsive breastfeeding and c) mother-infant interaction through early stimulation and learning through play (see Table 1 for description of intervention sessions).

The intervention is designed to be delivered by non-specialist facilitators in 15 monthly sessions in community settings and commences from third trimester of pregnancy and ends at 12-months postpartum. During the COVID-19 pandemic, when the in-person sessions are not possible, we are delivering the scheduled intervention session via telephone. All those sessions delivered via telephone are not repeated in-person.

Table 1: Description of intervention sessions

Sessions	Content	Timing	Recipients
1: Introduction and Rapport building	- Introducing program and engaging mother and her family<br>- Developing empathetic relationship with mother<br>- Ensuring family support system	Beginning of 7th month of pregnancy	Mother and family members
2: Maternal wellbeing	- Assessment of psychosocial problems of mother<br>- Use of guided discovery and behavioral activation for improving mother's health (strategies for improving overall well-being, including diet, rest, relaxation and sleep)	7th month of pregnancy	Mother and family members

For peer review only - http://bmjopen.bmj.com/site/about/guidelines.xhtml

3: Improving mother relationship with significant others	- Thought challenging and behavior activation using guided discovery (strategies for improving interpersonal relationships and mobilizing social support)<br>- Awareness about complication during pregnancy	8th month of pregnancy	Mother and family members
4: Improving mother relationship with infant during pregnancy	- Thought challenging and behavior activation using guided discovery (strategies for improving bond with the infant during pregnancy. knowledge and planning of safe delivery and importance of colostrum)	9th month of pregnancy	Mother
4: Follow-up session after delivery	- Following up after child birth<br>- Psycho-education about child development	Immediate post-delivery	Mother
5: Improving child's physical health	- Thought challenging and behavior activation using guided discovery (strategies for awareness on benefits of exclusive breastfeeding)<br>- Responsive breast feeding<br>- Addressing the concept of child crying because of insufficient milk<br>- Increasing breast milk by diet and improved frequency- addressing insufficiency of milk<br>- Family support for breast feeding<br>- Assessment of post-partum psychosocial problems of mother	8 weeks postnatal	Mother
5A: Improving maternal health	- Assessment of psychosocial problems of mother<br>- Use of guided discovery and behavioral activation for improving mother's health	9 weeks postnatal	Mother
5B: Improving maternal health	- Use of guided discovery and behavioral activation for improving mother's relationship with others	10 weeks postnatal	Mother
5C: Improving maternal health	- Use of guided discovery and behavioral activation for	11 weeks postnatal	Mother

For peer review only - http://bmjopen.bmj.com/site/about/guidelines.xhtml

	improving mother's relationship with child		
6: Importance of mother child interaction	- Exclusive Breast Feeding (EBF) for 6months<br>- Birth spacing<br>- Suitable environment- birth spacing<br>- Interaction, love and care for child<br>- Selecting play activity and conducting with mother and child	12 weeks postnatal	Mother and infant
7: Follow up on early stimulation activities	- Revision of activities discussed in session 6<br>- Importance of vaccination	16 weeks postnatal	Mother and Child
8: Awareness on birth spacing; improving child's health	Thought challenging and behavior activation using guided discovery (strategies for awareness on birth spacing and its relation to child's health)<br><br>- Exclusive breast feeding for 6-month<br>- Birth spacing<br>- Suitable environment- birth spacing<br>- Modern methods of contraception<br>- Interaction, love and care for child<br><br>Selecting play activity and conducting with mother and child	20 weeks postnatal	Mother
9: Introduction to solid food	- Interaction and play with child<br>- Selecting play activity and conducting with mother and child<br>- Introduction to Solid food and concept of super food<br>- Food and hygiene<br>- Continuation of breastfeeding with weaning at 6m<br>- Importance of vaccination	24 weeks postnatal	Mother
10: Introduction to mother child interaction.	- Skill based activities to promote mother-child interaction	28 weeks postnatal	Mother and infant

For peer review only - http://bmjopen.bmj.com/site/about/guidelines.xhtml

Improving mother child interaction through responsiveness and praise	- Homework charts are given to monitor the activity.<br>- Learning through play activities are reinforced<br>-		
11: Importance of following child's interest	- Skill based activities to promote mother-child interaction<br>- Homework charts are given to monitor the activity.<br>- Learning through play activities are reinforced	32 weeks postnatal	Mother and infant
12: Importance of following child's lead	- Skill based activities to promote mother-child interaction<br>- Homework charts are given to monitor the activity.<br>- Learning through play activities are reinforced	36 weeks postnatal	Mother and infant
13: Promote child language development	- Skill based activities to promote mother-child interaction<br>- Homework charts are given to monitor the activity.<br>- Learning through play activities are reinforced	40 weeks postnatal	Mother and infant
14: Scaffolding	- Skill based activities to promote mother-child interaction<br>- Homework charts are given to monitor the activity.<br>- Learning through play activities are reinforced	44 weeks postnatal	Mother and infant
15: Review of all 5 strategies	Revision of all strategies and next line of action.<br>Learning through play activities are reinforced	48 weeks postnatal	Mother and infant

For peer review only

Training and supervision of non-specialist facilitators in 'Nurturing Care' intervention

The delivery agents of the 'Nurturing Care' intervention are female non-specialist facilitators who are graduates in psychology/social sciences with little or no prior experience of delivering psychosocial interventions to mothers with perinatal distress and their new-born infants and have achieved competency to deliver 'Nurturing Care' intervention following training and practice sessions. These non-specialist facilitators were selected from the universities in the study sub-district through a job advertisement and interviews.

A cascade model of training and supervision has been followed to deliver 'Nurturing Care' intervention. The training consisted of classroom teaching of basic theoretical concepts of 'Nurturing Care' intervention, its strategies, group discussions and role-plays. The program

For peer review only - http://bmjopen.bmj.com/site/about/guidelines.xhtml

trainers trained 15 non-specialist facilitators. The training consisted of 10-days class-room training followed by practice cases under the supervision of program trainer. The participants of this practice group were pregnant women without distress and their infants, selected from the same community after seeking informed consent for participation in the training. The program trainer performed live competency rating during practice cases using an adapted version of ENhancing Assessment of Common Therapeutic factors (ENACT) tool for 'Nurturing Care' intervention (Kohrt et al. 2015). Only competent non-specialist facilitators (mean score above 2.5 on all domains of adapted ENACT) have been allowed to deliver 'Nurturing Care' intervention. Each non-specialist facilitator has been assigned a case load of 8-10 mothers to deliver intervention. The fidelity of the intervention is being evaluated using an adapted ENACT tool developed for the program. 20% of randomly selected intervention sessions from each non-specialist facilitators at different stages of the intervention delivery will be rated by the program trainers to assess the fidelity of intervention delivery by non-specialist facilitators.

Supervision

Program trainers are experts in perinatal mental health and infant development and provide weekly supervision to non-specialist facilitators to ensure intervention fidelity. Supervision involves discussion of difficulties encountered in delivering intervention delivery and, as well as self-care of the non-specialist facilitators.

Treatment as Usual (TAU)

As a part of treatment-as-usual, participants in both study arms receive routine visits by the Lady Health Workers (LHWs) of their respective areas. The LHWs are trained to provide preventive maternal and child care including psycho-education about family planning and maternal and child health; provide free of cost contraceptives and antenatal-iron-folic acid supplements throughout the pregnancy starting from second trimester via door to door visits to the households of their allocated areas. LHWs visit new mothers on a monthly basis to monitor the progress of a newborn baby on growth parameters such as weight and height. Moreover, they are given additional duties (provide information about infectious disease or to administer oral polio drops to children) which requires regular visits to houses. In coordination with traditional birth attendants and affiliated nearest health facility, LHWs also provide referral service to mothers for safe motherhood. In the current study, the number of visits mothers receive by LHWs will be the same for each trial participant regardless of their allocation status.

If, during the study's assessments time-points participants in both arms show severe psychiatric disorders that require immediate specialist treatment and follow-up, they will be referred to tertiary mental health care facility, Institute of Psychiatry, Benazir Bhutto Hospital, Rawalpindi. A complete record of services received by trial participants in both arms will be made using the Client Services Receipt Inventory (CSRI).

Outcomes measures

The effectiveness of intervention will be evaluated on a range of caregiver and child outcomes. The outcome measures will be administered by the trained assessment team blind to the allocation status of participants at baseline (at the time of recruitment i.e. 3rd trimester of pregnancy), 6 and 12-months' postpartum. The self-report outcome measures will be orally administered by the trained assessment team blind to the allocation status of the participants. The detail of the outcome measures is given below (see Table 2 for schedule of assessments);

Primary Outcome

For peer review only - http://bmjopen.bmj.com/site/about/guidelines.xhtml

Caregivers Child Interaction (CCI): Maternal responsive caregiving behaviours will be assessed at 12-months' postpartum across two-arms (primary endpoint) using the **Observation of Mother and Child Interaction (OMCI) tool [22].** OMCI tool is based on responsive parenting framework proposed by Landry and colleagues [23]. The tool comprises of 19 items related to maternal and child behaviors. Maternal behaviors include maternal affect, maternal touch, maternal verbalization, sensitivity and contingent responses, scaffolding, language stimulation, and focus; whereas, child outcomes include, child affect, child focus, child's communication efforts, and mutual enjoyment. The scoring is done by recording the frequency of the occurrence of each of the above mentioned behaviors during the mother-child interaction. The sum is calculated by adding the scores of mother and child domains; where higher score indicates more responsive interactions. The tool has been designed to live rate mother-child interaction of 2-years old toddlers in low resource settings of rural Pakistan. It has been extensively used in similar population in Pakistan and found to be sensitive to detect change in mother-child interaction as a result of receiving responsive caregivers intervention at 12 and 24-months post-partum [22]. In the present study, the research assistants will observe a live 5-minute mother-infant interaction while mother and infant will play together with a picture book.

Infant secondary outcomes

1. **Bayley's Scales of Infant Development (BSID III**) [24] will be used to measure cognitive, language, motor, socio-emotional development and adaptive behaviour at 12-months' postpartum. The tool has been used previously in similar South Asian settings [25, 26].
2. **Ages and Stages Questionnaire 3rd Edition (ASQ3) [27]** will be used to measure developmental delays in communication, gross motor, fine motor, problem solving, and personal social domains at 12-months' postpartum. It provides cut-off score in 5 domains of development that indicates possible need for further evaluation. It highlights results that fall in a "monitoring zone," to make it easier to keep track of children at-risk. The tool has been validated to use in South Asian community settings [26, 28].
3. Home environment measured by **Home inventory [29]:** Quality and quantity of stimulation and support in the child's home will be measured using home inventory at 12-months' postpartum. The tool has been used validated to use in Pakistan [30].
4. **Anthropometric measurements [31]**: Infants in both groups will be weighed and measured with standard techniques at 12-months' postpartum. Growth data will be converted into SDs (Z scores) for weight and length with WHO Anthro (version 3.2.2).
5. **Early breastfeeding (EBF) [32]**: Assessors will document what the infant was fed in the last 24 hours at 6 months postpartum. This information will be categorized as either EBF, partial breastfeeding (giving an infant some breastfeeding, and some additional foods, either milk, cereal, or other food items), or no breastfeeding. If the infant will not exclusively breastfeed, details of when (age of infant) and why EBF was discontinued will be recorded. At the first 6-months follow-up, details about early initiation, discarding colostrum, use of pre-lacteals, and reasons for delaying or not initiating breastfeeding will be assessed.
6. The number of **diarrhoeal episodes** in the infants during the 2 weeks before interview will be recorded at 6 and 12-months' postpartum, with a questionnaire used in previous studies [33]. Diarrhoea will be defined as three or more unformed stools passed in 24 hours, and a diarrhoeal episode will be defined as being separated from another episode by at least 3 diarrhoea-free days.

For peer review only - http://bmjopen.bmj.com/site/about/guidelines.xhtml

7. Records of **immunisation** will be assessed for all infants in the study, and infants will be classified as those with or without up-to-date immunisation status 6 and 12-months' postpartum.

Maternal secondary outcomes;

8. Maternal distress will be measured by **the Self Reporting Questionnaire (SRQ**) [34] at baseline, 6 and 12-months postpartum. SRQ is a 20-item self-report measure to detect non-specific psychological distress, developed by the World Health Organization (WHO). Psychological distress is represented by subscales of depression/anxiety, somatic symptoms, reduced vital energy and depressive thoughts. The SRQ items are scored 0 or 1. A score of 1 indicates the presence of symptoms of psychological distress during past month and a score of 0 indicates absence of symptoms. The maximum score indicates presence of higher psychological distress. A score of > 9 indicates increased risk of maternal distress/post-partum depression [8, 35].
9. Caregivers' health related quality of life will be measured by **WHO Quality of Life (WHOQoL)- Brief version** at baseline and 12-months postpartum. WHOQoL assess the participants position in life in the context of the culture and value systems in which they live and in relation to their goals, expectations, standards and concerns. WHOQoL comprises of 4 domains namely physical health, psychological health, social relationships and environment. Items are rated on a 5-point Likert scale, where 1 represents "disagree" or "not at all" and 5 represents "completely agree" or "extremely".
10. The level of social support will be measured using **Multi-Dimensional Scale of Perceived Social Support (MSPSS) [36]** will be measured baseline and 12-months' postpartum. It includes 12 items which cover three dimensions: support from family, friends and significant other. Each item is rated on a 7-point Likert-scale (1 = *very strongly disagree*; 7 = *very strongly agree*). A total score is calculated by summing the responses of all items (range 12–84) with higher scores indicating higher levels of perceived social support.
11. Caregiver's level of functioning will be measured using **World Health Organization Disability Assessment Schedule (WHODAS) [37]** at baseline and 12-months' post-partum. WHODAS assesses participants' health-related difficulties in the level of functioning in six domains of life (understanding and communicating; moving and getting around; attending to one's hygiene, dressing, eating and staying alone; interacting with other people; domestic responsibilities, leisure, work and school and joining in community activities, participating in society) over the past 30 days.
12. **Maternal Financial empowerment**: Women's empowerment will be measured at baseline and 12-months postpartum, using a questionnaire consisting of: (a) two items previously used in the same population [38] that assess financial empowerment (i.e., whether a woman is given a lump-sum of money for day-to-day expenses and whether she can make independent decisions about its use). Women who answer '*yes*' to both items on financial empowerment will be classified as 'empowered'.
13. Utilization of health care will be measured by **Client Service Receipt Inventory (CSRI)** adapted for perinatal population of Pakistan, at 12 months postpartum [32].

For peer review only - http://bmjopen.bmj.com/site/about/guidelines.xhtml

Table 2: Schedule of assessments

Sr. no.	Outcomes		Time points		
		Screening	Baseline 3rd trimester of pregnancy	6-months post-partum	12 months postpartum
Primary outcome					
1.	OMCI				X
Secondary outcomes					
2.	SRQ	X	X	X	X
3.	WHO-QoL		X		X
4.	MSPSS		X		X
5.	WHODAS		X		X
6.	Maternal empowerment-brief questionnaire		X		X
7.	BSID-III				X
8.	ASQ3				X
9.	Home inventory				X
10.	Weight and height measurements				X
11.	Early breastfeeding-brief questionnaire			X	
12.	Diarrhoeal episodes -brief questionnaire			X	X
13.	Immunization-record form			X	X
14.	CSRI				X

OMCI, Observation of Mother-Child Interaction; SRQ, Self-reporting Questionnaire; WHO-QoL, World Health Organization, Quality of Life; MSPSS, Multi-Dimensional Scale of Perceived Social Support; WHODAS, WHO Disability Assessment Schedule; BSID-III, Brief questionnaire, Bayley Scale of Infant Development-III; ASQ3, Ages and Stages Questionnaire-3; CSRI, Client Service Receipt Inventory

As the study is being conducted amidst COVID-19, all in-person assessments will be conducted by following appropriate precautionary measures.

Randomization and blinding

The participants were randomized into intervention or control arm on a 1:1 basis using computerized software by an independent researcher. The allocation concealment was ensured by keeping random sequence in sequentially numbered, opaque, sealed envelopes, at the off-site centre. Only assessment team (conducting baseline and follow-up assessment) will be blind to the treatment allocation of trial participants. To maintain the blinding, all trial participants will be briefed, prior to the assessment about the significance of blinding and the importance of not disclosing their allocation status to the assessment team members. If un-blinding does occur, this will be documented, the assessment will be halted and a new assessor will be assigned to complete the assessment.

Ethical considerations

For peer review only - http://bmjopen.bmj.com/site/about/guidelines.xhtml

For peer review only

Ethical approval for the study was obtained from Institutional Review Board (IRB) of Human Development Research Foundation (HDRF). Informed consent was obtained from all participants prior to data collection. Privacy and confidentiality of the research participants will be ensured throughout the study.

Other ethical considerations

In the present study, a referral pathway, based on the existing referral system, has been established to ensure the safety of the study participants. The Institute of Psychiatry (IoP) is the tertiary mental health care facility in the north of Pakistan and situated within the public tertiary healthcare facility and teaching hospital, the Benazir Bhutto Hospital, Rawalpindi. It caters to the mental health needs of the populations in the metropolitan city of Rawalpindi and is the specialist referral facility for common mental health problems identified at the adjacent primary healthcare centers. In our study, if a participant discloses any event of abuse, intimate partner violence, or is at imminent risk of harm to herself and others, identified at the time of assessment or during the intervention delivery, the research team will record the event and referral to the IoP will be made for appropriate medical, medico-legal and psychosocial assessment and rehabilitation by a multi-disciplinary team led by qualified psychiatrists.

Adverse events reporting

All adverse events (AEs) and serious adverse events (SAEs) reported by the trial participants, their husband and in-laws of recruited participants, LHWs or observed by the investigator or the research team (assessment & intervention team) will be recorded by data manager. AE is defined as any undesirable experience occurring to a subject during the time frame of the study, whether or not considered relevant to the research procedure. All AEs will be collected by assessment team at 12-months' postpartum follows and will be reviewed periodically by trial steering committee which will determine the necessary steps to be taken with respect to the ongoing trial.

Data management

The baseline and follow-up assessments data will be collected by the assessment team on android based application using hand held devices (tablets). Data will be stored at the secure web server and will be checked for consistency & quality at the end of each day. All data sets will be stored in password protected computer, only accessible by the responsible individual. The data collected in hard copies (collected by the interviewers) will be safely stored in locked cabinets. Qualitative data will be fully anonymized and coded. No identifying names or details will be recorded.

Statistical analysis

The findings will be reported according to the CONSORT guidelines for RCT [39] (Figure 1). This will include the flow of research participants through each stage of the trial, including the number eligible, randomly assigned, receiving the intended treatment, completing the study protocol and analysed for the primary outcome. The primary analyses will be on intent-to-treat basis consisting of all participants included according to the arms in which they are randomized and secondary analyses will be based on per-protocol population.

For the analysis of the primary outcome (caregiver-child interaction across two-arms at 12-months post-partum), a linear regression model will be employed with treatment as the sole predictor. In addition, adjusted linear model analysis will be performed with the pre-specified covariates (gender of infant, mother's education and severity of mothers' psychosocial distress measured using SRQ) measured at baseline being added into the above linear model. The crude and adjusted

For peer review only - http://bmjopen.bmj.com/site/about/guidelines.xhtml

mean differences in the primary outcome together with its 95% confidence intervals at 12-months will be derived from the linear models. In addition, subgroup analysis of primary endpoint will be performed on the above pre-specified covariates.

Analysis of secondary continuous outcomes with single follow-up measurement will be done in a similar fashion as the primary endpoint analysis. Analysis of secondary continuous outcomes with repeated follow-up measurement will be performed using a linear mixed model with treatment visit, interaction between treatment and visit as fixed effects, the baseline value of the outcome as covariate, if it is available and subject as random effects. The analysis of binary outcomes will also use a generalized linear/mixed model depending on whether there will be a repeated measurement. Odds ratios with their 95% confidence intervals will be derived from the generalized linear/mixed model analysis. Missing primary outcome and secondary outcomes with a single measurement will not be imputed; whereas, missing secondary outcomes with repeated measurements will be imputed in sensitivity analysis using the last observation carried forward strategy. Missing baseline covariates will be imputed using simple imputation methods in the covariate adjusted analysis based on the covariate distributions in the sample. For a continuous variable, missing values will be imputed from random values from a normal distribution with mean and SD calculated from the available sample. For a categorical variable, missing values will be imputed from random values from a uniform distribution with probabilities P_1, P_2, …, and P_k from the sample. Seed for the imputation will be set as the date of data analysis. The study is powered to make a single comparison in the primary outcome at 12 months only. All analyses will be described in detail in the statistical analysis plan. SAS 9.4 and SPSS 21 will be employed for the statistical analyses.

Potential mediators

We will explore pathways through which the intervention may influence primary outcome (i.e. mother-child interaction). There is strong empirical evidence that the quality of the mother-child interactions, such as her sensitivity and responsiveness, is associated with both maternal depression and child development [40-43]. there is also some evidence that perinatal depression interventions, especially ones that include a parenting component, can improve maternal distress; maternal responsiveness, resulting in improved developmental outcomes [44]. We will assess maternal responsiveness with the maternal items of OMCI that measure maternal responsive behaviors during mother-child interaction. The OMCI has been used to evaluate the impact of maternal interventions on maternal responsiveness [45]. Maternal distress will be assessed using SRQ. The tool has been used extensively in the study settings previously to measure maternal distress [46].

Social support: Low social support is a consistent predictor of mental ill health among mothers [47, 48]. Social support can be theorized to be either a mediator or effect modifier; since building social support is a key feature of the integrated intervention, we hypothesize that increased social support will mediate the relationship between the intervention and child outcomes. *Maternal empowerment*: Maternal empowerment will be assessed using two items previously used in the same population [38]. *Breastfeeding and other health related variables*; Perinatal depression interventions similar to this integrated intervention have resulted in higher rates of exclusive breastfeeding at 6 months and increased likelihood of completion of scheduled immunizations [6]. These health-related behaviours may contribute to the positive effect of intervention on improving maternal wellbeing and infant development, we will assess the impact of behaviours related to feeding (breastfeeding, introduction of solid food) and immunizations on mother-child interaction.

For peer review only - http://bmjopen.bmj.com/site/about/guidelines.xhtml

Cost-effectiveness analysis

Health economic analysis will be conducted to determine the difference in costs and outcomes in the intervention as compared to the control arm. The cost of the intervention will be calculated based on the sum of materials, personnel training, and delivery. We will then compare the combination of the cost of the intervention and the service cost data with mother-child interaction summary scores. If the intervention results in more favourable summary scores (improvement in mother-infant interaction) and lower total costs, it will be described as superior to no intervention option. If, in contrast, the intervention results in more favourable scores but is also more expensive, then the cost-effectiveness calculation will be based on the cost and scores of mother-infant interaction outcomes.

Primary analysis will be of total costs over the 12-months post-partum follow-up treatment period. Recognizing that cost data are often skewed, the bootstrap technique will be applied. The sampling with replacement from original observed pairs of costs and effects will be employed to maintain correlation structure between costs and benefits, and bootstrapping sampling will be repeated 1000 times. For each bootstrap sample, an estimate of differential total mean costs and expected mean effectiveness will be calculated. The 95% CIs for the differential estimates will be derived from the 25th and 97.5^{th} percentiles [49, 50].

Cost-effectiveness will be assessed by combing costs with the primary outcome measure (i.e. mother-infant interaction) scores in incremental cost-effectiveness analysis. Repeat resampling from the costs and effectiveness data (bootstrapping) will be used to calculate the probability that each of the treatment is the optimal choice, subject to a range of possible maximum values (ceiling ratio) that a decision-maker might be willing to pay for a unit improvement in mother-infant interaction scores. The results of the cost-effectiveness will be reported as incremental cost-effectiveness ratios, and acceptability curves which summarise the information contained in a cost-effectiveness plane [51].

Mixed-methods evaluation

We will evaluate the feasibility of our intervention program using mixed methods study design. Mixed-methods evaluation of implementation will be carried out to assess assumptions underlying the intervention strategy and will cover other aspects of program implementation such as number of research participants who signed-up and complete the intervention and satisfaction with intervention, difficulties and successes in carrying out intervention activities, acceptability, feasibility and appropriateness of using integrated intervention.

Satisfaction with the intervention and barriers and facilitators to implementation, feasibility of workload, and difficulties and successes in carrying out intervention activities and satisfaction with training and supervision will be explored through semi-structured interviews with the relevant stakeholders.

Trial management

Trial management will be provided by Trial Steering Committee (TSC) that comprises Principal Investigator (PI), Co-Investigator (s), trial coordinator, senior researchers and intervention staff who will meet monthly.

Dissemination

There is a growing evidence base on the feasibility, acceptability and effectiveness of peer/parent delivered, psychosocial and early child development interventions [6, 18, 52]. Training and supervision of large numbers of health workers in new interventions is a key barrier to scale up of programs. Our intervention combines evidence-based elements into a single package, making delivery more effective and synergistic, through a single delivery agent i.e. non-specialist

For peer review only - http://bmjopen.bmj.com/site/about/guidelines.xhtml

facilitator. The use of non-specialist facilitators in disseminating the interventions has the potential to extend the reach of the intervention in a cost-effective fashion. If proven effective, the study will contribute to scale-up care for maternal and child mental health in low resource settings globally. We will disseminate our results to key stakeholders. Dissemination activities will include publication of study results in open access, peer-reviewed journals, and presentations at academic conferences and community forums.

Ethics and dissemination

Ethics approval for the present study was obtained from the Human Development Research Foundation (HDRF) Institutional Review Board, Islamabad Pakistan. The findings of the present study will be published in peer-reviewed journals and presented at conferences and community forums.

References:

1. Black, R., et al., *Maternal and child undernutrition and overweight in low-income and middle-income countries.* The lancet, 2013. **382**(9890): p. 427-451.
2. Grantham-McGregor, S., et al., *Child Development in developing countries 1: Developmental potential in the first 5 years for children in developing countries.* Lancet, 2007(369): p. 60-70.
3. Fisher, J., et al., *Prevalence, nature, severity and correlates of postpartum depressive symptoms in Vietnam.* BJOG: An International Journal of Obstetrics & Gynaecology, 2004. **111**(12): p. 1353-1360.
4. Rahman, A., et al., *Impact of maternal depression on infant nutritional status and illness - A cohort study.* Archives of General Psychiatry, 2004. **61**(9): p. 946-952.
5. Hurley, K.M., A.K. Yousafzai, and F. Lopez-Boo, *Early child development and nutrition: a review of the benefits and challenges of implementing integrated interventions.* J Advances in nutrition, 2016. **7**(2): p. 357-363.
6. Rahman, A., et al., *Cognitive behaviour therapy-based intervention by community health workers for mothers with depression and their infants in rural Pakistan: a cluster-randomized controlled trial.* The Lancet, 2008. **372**(9642): p. 902-909.
7. Yousafzai, A.K., et al., *Effect of integrated responsive stimulation and nutrition interventions in the Lady Health Worker programme in Pakistan on child development, growth, and health outcomes: a cluster-randomized factorial effectiveness trial.* The Lancet, 2014. **384**(9950): p. 1282-1293.
8. Yousafzai, A.K., et al., *Effects of responsive stimulation and nutrition interventions on children's development and growth at age 4 years in a disadvantaged population in Pakistan: a longitudinal follow-up of a cluster-randomized factorial effectiveness trial.* The Lancet Global Health, 2016. **4**(8): p. e548-e558.
9. Stevens, G.A., et al., *Trends in mild, moderate, and severe stunting and underweight, and progress towards MDG 1 in 141 developing countries: a systematic analysis of population representative data.* 2012. **380**(9844): p. 824-834.
10. Rahman, R. Harrington, and J. Bunn, *Can maternal depression increase infant risk of illness and growth impairment in developing countries?* Journal of Child Care, health and development 2002. **28**(1): p. 51-56.
11. Glover, V., *Maternal depression, anxiety and stress during pregnancy and child outcome; what needs to be done.* Best Pract Res Clin Obstet Gynaecol, 2014. **28**(1): p. 25-35.

For peer review only - http://bmjopen.bmj.com/site/about/guidelines.xhtml

12. Hamdani, S.U., et al., *Family networks to improve outcomes in children with intellectual and developmental disorders: a qualitative study.* J International journal of mental health systems, 2014. **8**(1): p. 7.
13. Busch-Hallen, J., et al., *Impact of COVID-19 on maternal and child health.* Lancet Glob Health, 2020. **8**(10): p. e1257.
14. Chorpita, B.F., E.L. Daleiden, and J.R. Weisz, *Identifying and selecting the common elements of evidence based interventions: A distillation and matching model.* Mental health services research, 2005. **7**(1): p. 5-20.
15. UNICEF. *Care for Child Development Package*. 2012; Available from: https://www.unicef.org/earlychildhood/index_68195.html.
16. Salomone, E., et al., *Development of the WHO Caregiver Skills Training Program for Developmental Disorders or Delays.* 2019. **10**(769).
17. WHO. *Thinking Healthy*. 2015 [cited 2020 Sep 16]; Available from: https://www.who.int/mental_health/maternal-child/thinking_healthy/en/.
18. Zafar, S., et al., *Integrating maternal psychosocial well-being into a child-development intervention: the five-pillars approach.* Ann N Y Acad Sci, 2014. **1308**: p. 107-17.
19. NIPS. *Pakistan demographic and health survey 2012–13*. 2013 [cited 2015 April 15]; Available from: https://dhsprogram.com/pubs/pdf/fr290/fr290.pdf.
20. Rahman, A. and F. Creed, *Outcome of prenatal depression and risk factors associated with persistence in the first postnatal year: Prospective study from Rawalpindi, Pakistan.* Journal of Affective Disorders, 2007. **100**(1-3): p. 115-121.
21. Bureau of Statistics and UNICEF Pakistan, *District based Multiple Indicator Cluster Survey (MICS), 2003-2004: Punjab*. 2003, Government of Punjab, Planning and Development Department, Federal Bureau of Statistics Islamabad United Nations Children's Fund, 2004: Lahore
22. Rasheed, M.A. and A.K. Yousafzai, *The development and reliability of an observational tool for assessing mother–child interactions in field studies‐experience from Pakistan.* Child: care, health and development, 2015. **41**(6): p. 1161-1171.
23. Landry, S.H., et al., *Does early responsive parenting have a special importance for children's development or is consistency across early childhood necessary?* Developmental psychology, 2001. **37**(3): p. 387.
24. Albers, C.A. and A.J. Grieve, *Test review: Bayley, N.(2006). Bayley scales of infant and toddler development–third edition. San Antonio, TX: Harcourt assessment.* Journal of Psychoeducational Assessment, 2007. **25**(2): p. 180-190.
25. Kvestad, I., et al., *The assessment of developmental status using the Ages and Stages questionnaire-3 in nutritional research in north Indian young children.* Nutr J, 2013. **12**: p. 50.
26. Sikander, S., et al., *Cohort Profile: Perinatal depression and child socioemotional development ; the Bachpan cohort study from rural Pakistan.* BMJ Open, 2019. **9**(5): p. e025644.
27. Squires, J., D.D. Bricker, and E. Twombly, *Ages & stages questionnaires*. 2009: Paul H. Brookes Baltimore, MD.
28. Bhopal, S.S., et al., *Using the Mothers Object Relations Scale for early childhood development research in rural India: Findings from the Early Life Stress Sub-study of the SPRING Cluster Randomized Controlled Trial (SPRING-ELS).* Journal of Wellcome Open Research, 2021. **6**(54): p. 54.

For peer review only - http://bmjopen.bmj.com/site/about/guidelines.xhtml

29. Caldwell, B. and R. Bradley, *Home observation for measurement of the environment*. 1984: University of Arkansas at Little Rock Little Rock.
30. Yousafzai, A.K., et al., *Parenting Skills and Emotional Availability: An RCT.* Pediatrics, 2015. **135**(5): p. e1247-57.
31. WHO Expert Committee, *Physical status: the use and interpretation of anthropometry.* http://helid. digicollection. Org/en/d/Jh0211e/, 1995.
32. Sikander, S., et al., *Cognitive-behavioral counseling for exclusive breastfeeding in rural pediatrics: a cluster RCT.* Pediatrics, 2015: p. peds. 2014-1628.
33. Rahman, A., et al., *Maternal depression increases infant risk of diarrhoeal illness:–a cohort study.* Archives of Disease in Childhood, 2007. **92**(1): p. 24-28.
34. Beusenberg, M., J.H. Orley, and W.H. Organization, *A User's guide to the self reporting questionnaire (SRQ*. 1994, Geneva: World Health Organization.
35. Santos, I.S., et al., *Comparing validity of Edinburgh scale and SRQ20 in screening for post-partum depression.* Clinical practice and epidemiology in mental health : CP & EMH, 2007. **3**: p. 18-18.
36. Dahlem, N.W., G.D. Zimet, and R.R. Walker, *THE MULTIDIMENSIONAL SCALE OF PERCEIVED SOCIAL SUPPORT - A CONFIRMATION STUDY.* Journal of Clinical Psychology, 1991. **47**(6): p. 756-761.
37. Üstün, T.B., et al., *Developing the World Health Organization disability assessment schedule 2.0.* Bulletin of the World Health Organization, 2010. **88**: p. 815-823.
38. Rahman, A., et al., *Effective treatment of perinatal depression for women in debt and lacking financial empowerment in a low-income country.* The British Journal of Psychiatry, 2012. **201**(6): p. 451-457.
39. Moher, D., et al., *CONSORT 2010 Explanation and Elaboration: updated guidelines for reporting parallel group randomized trials.* 2010. **340**: p. c869.
40. Appelbaum, M., et al., *The effects of infant child care on infant-mother attachment security: Results of the NICHD study of early child care.* Child Development, 1997. **68**(5): p. 860-879.
41. Black, M., et al., *Depressive symptoms among rural Bangladeshi mothers: implications for infant development.* Journal of Child Psychology and Psychiatry, 2007. **48**(8): p. 764-772.
42. Schmid, B., et al., *Quality of early mother-child interaction associated with depressive psychopathology in the offspring: A prospective study from infancy to adulthood.* Journal of Psychiatric Research, 2011. **45**(10): p. 1387-1394.
43. Tamis-LeMonda, C.S., M.H. Bornstein, and L. Baumwell, *Maternal responsiveness and children's achievement of language milestones.* Child Development, 2001. **72**(3): p. 748-767.
44. van Doesum, K.T.M., et al., *A randomized controlled trial of a home-visiting intervention aimed at preventing relationship problems in depressed mothers and their infants.* Child Development, 2008. **79**(3): p. 547-561.
45. Beeber, L.S., et al., *Short-Term In-Home Intervention Reduces Depressive Symptoms in Early Head Start Latina Mothers of Infants and Toddlers.* Research in Nursing & Health, 2010. **33**(1): p. 60-76.
46. Rahman, A., *Screening for postnatal depression in the developing world: a comparison of the WHO Self-Reporting Questionnaire (SRQ-20) and the Edinburgh Postnatal Depression Screen (EPDS).* J Pak Psychiatr Soc, 2005. **2**: p. 69-72.

For peer review only - http://bmjopen.bmj.com/site/about/guidelines.xhtml

47. Fisher, J., et al., *Prevalence and determinants of common perinatal mental disorders in women in low- and lower-middle-income countries: a systematic review.* Bulletin of the World Health Organization, 2012. **90**(2): p. 139-149.

48. Rahman, A., Z. Iqbal, and R. Harrington, *Life events, social support and depression in childbirth: perspectives from a rural community in the developing world.* Psychological Medicine, 2003. **33**(7): p. 1161-1167.

49. Barber, J.A. and S.G. Thompson, *Analysis and interpretation of cost data in randomized controlled trials: review of published studies.* BMJ, 1998. **317**(7167): p. 1195-1200.

50. Efron, B. and R. Tibshirani, *An Introduction to the Bootstrap, vol. 23, no. 2.* 1998, Chapman & Hall, New York.

51. Fenwick, E., K. Claxton, and M. Sculpher, *Representing uncertainty: the role of cost‐effectiveness acceptability curves.* Health economics, 2001. **10**(8): p. 779-787.

52. Hamdani, S.U., et al., *WHO Parents Skills Training (PST) programme for children with developmental disorders and delays delivered by Family Volunteers in rural Pakistan: study protocol for effectiveness implementation hybrid cluster randomized controlled trial.* Global mental health, 2017. **4**.

Acknowledgment

The study sponsor and funders have no role in the study design; collection, management, analysis, and interpretation of data; writing of the report; and the decision to submit the report for publication.

Authors' Contributors

SUH, ZeH & DW will have the access of all datasets. ZeH drafted the initial manuscript. SUH conceived the study and wrote the final manuscript. AG, FS, AR, BM, HJ, DW & AR contributed to the writing. All authors approved of the final version of the manuscript.

Funding statement

This work is supported by Grand Challenges Canada, Saving Brains, Grant Number SB-POC-1809-18680

Competing interests statement None declared.

Word count 5442 (without Tables and Figure)

For peer review only

For peer review only - http://bmjopen.bmj.com/site/about/guidelines.xhtml

Fig 1: Flow Diagram

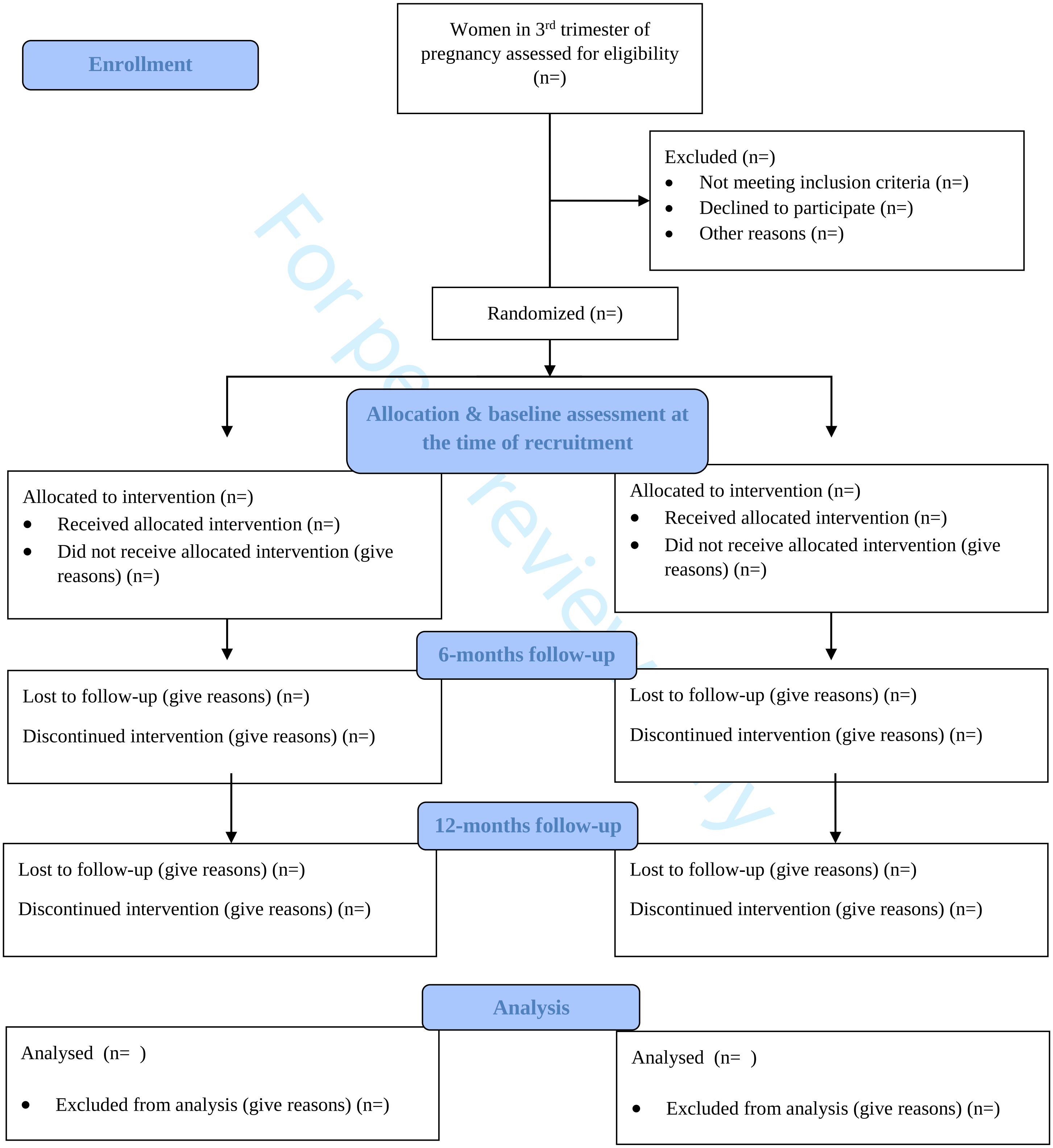

For peer review only - http://bmjopen.bmj.com/site/about/guidelines.xhtml

STANDARD PROTOCOL ITEMS: RECOMMENDATIONS FOR INTERVENTIONAL TRIALS

SPIRIT 2013 Checklist: Recommended items to address in a clinical trial protocol and related documents*

Section/item	Item No	Description	Addressed on page no.
**Administrative information**			
Title	1	Descriptive title identifying the study design, population, interventions, and, if applicable, trial acronym	Title p.1
Trial registration	2a	Trial identifier and registry name. If not yet registered, name of intended registry	2
	2b	All items from the World Health Organization Trial Registration Data Set	Included in the registration
Protocol version	3	Date and version identifier	N/A
Funding	4	Sources and types of financial, material, and other support	18
Roles and responsibilities	5a	Names, affiliations, and roles of protocol contributors	1 & 18
	5b	Name and contact information for the trial sponsor	N/A
	5c	Role of study sponsor and funders, if any, in study design; collection, management, analysis, and interpretation of data; writing of the report; and the decision to submit the report for publication, including whether they will have ultimate authority over any of these activities	18
	5d	Composition, roles, and responsibilities of the coordinating centre, steering committee, endpoint adjudication committee, data management team, and other individuals or groups overseeing the trial, if applicable (see Item 21a for data monitoring committee)	15
**Introduction**			
Background and rationale	6a	Description of research question and justification for undertaking the trial, including summary of relevant studies (published and unpublished) examining benefits and harms for each intervention	3

For peer review only - http://bmjopen.bmj.com/site/about/guidelines.xhtml

	6b	Explanation for choice of comparators	9
Objectives	7	Specific objectives or hypotheses	3
Trial design	8	Description of trial design including type of trial (eg, parallel group, crossover, factorial, single group), allocation ratio, and framework (eg, superiority, equivalence, noninferiority, exploratory)	3
**Methods: Participants, interventions, and outcomes**			
Study setting	9	Description of study settings (eg, community clinic, academic hospital) and list of countries where data will be collected. Reference to where list of study sites can be obtained	4
Eligibility criteria	10	Inclusion and exclusion criteria for participants. If applicable, eligibility criteria for study centres and individuals who will perform the interventions (eg, surgeons, psychotherapists)	4
Interventions	11a	Interventions for each group with sufficient detail to allow replication, including how and when they will be administered	5-9
	11b	Criteria for discontinuing or modifying allocated interventions for a given trial participant (eg, drug dose change in response to harms, participant request, or improving/worsening disease)	N/A
	11c	Strategies to improve adherence to intervention protocols, and any procedures for monitoring adherence (eg, drug tablet return, laboratory tests)	N/A
	11d	Relevant concomitant care and interventions that are permitted or prohibited during the trial	9
Outcomes	12	Primary, secondary, and other outcomes, including the specific measurement variable (eg, systolic blood pressure), analysis metric (eg, change from baseline, final value, time to event), method of aggregation (eg, median, proportion), and time point for each outcome. Explanation of the clinical relevance of chosen efficacy and harm outcomes is strongly recommended	10-12
Participant timeline	13	Time schedule of enrolment, interventions (including any run-ins and washouts), assessments, and visits for participants. A schematic diagram is highly recommended	12 & 19

For peer review only

		(see Figure)	
Sample size	14	Estimated number of participants needed to achieve study objectives and how it was determined, including clinical and statistical assumptions supporting any sample size calculations	4 & 5
Recruitment	15	Strategies for achieving adequate participant enrolment to reach target sample size	N/A
**Methods: Assignment of interventions (for controlled trials)**			
Allocation:			
Sequence generation	16a	Method of generating the allocation sequence (eg, computer-generated random numbers), and list of any factors for stratification. To reduce predictability of a random sequence, details of any planned restriction (eg, blocking) should be provided in a separate document that is unavailable to those who enrol participants or assign interventions	12
Allocation concealment mechanism	16b	Mechanism of implementing the allocation sequence (eg, central telephone; sequentially numbered, opaque, sealed envelopes), describing any steps to conceal the sequence until interventions are assigned	12
Implementation	16c	Who will generate the allocation sequence, who will enrol participants, and who will assign participants to interventions	12
Blinding (masking)	17a	Who will be blinded after assignment to interventions (eg, trial participants, care providers, outcome assessors, data analysts), and how	12
	17b	If blinded, circumstances under which unblinding is permissible, and procedure for revealing a participant's allocated intervention during the trial	12
**Methods: Data collection, management, and analysis**			
Data collection methods	18a	Plans for assessment and collection of outcome, baseline, and other trial data, including any related processes to promote data quality (eg, duplicate measurements, training of assessors) and a description of study instruments (eg, questionnaires, laboratory tests) along with their reliability and validity, if known. Reference to where data collection forms can be found, if not in the protocol	10-12
	18b	Plans to promote participant retention and complete follow-up, including list of any outcome data to be collected for participants who discontinue or deviate from	N/A

For peer review only

		intervention protocols	
Data management	19	Plans for data entry, coding, security, and storage, including any related processes to promote data quality (eg, double data entry; range checks for data values). Reference to where details of data management procedures can be found, if not in the protocol	13
Statistical methods	20a	Statistical methods for analysing primary and secondary outcomes. Reference to where other details of the statistical analysis plan can be found, if not in the protocol	13
	20b	Methods for any additional analyses (eg, subgroup and adjusted analyses)	14-15
	20c	Definition of analysis population relating to protocol non-adherence (eg, as randomised analysis), and any statistical methods to handle missing data (eg, multiple imputation)	13
**Methods: Monitoring**			
Data monitoring	21a	Composition of data monitoring committee (DMC); summary of its role and reporting structure; statement of whether it is independent from the sponsor and competing interests; and reference to where further details about its charter can be found, if not in the protocol. Alternatively, an explanation of why a DMC is not needed	15
	21b	Description of any interim analyses and stopping guidelines, including who will have access to these interim results and make the final decision to terminate the trial	N/A
Harms	22	Plans for collecting, assessing, reporting, and managing solicited and spontaneously reported adverse events and other unintended effects of trial interventions or trial conduct	12-13
Auditing	23	Frequency and procedures for auditing trial conduct, if any, and whether the process will be independent from investigators and the sponsor	N/A
**Ethics and dissemination**			
Research ethics approval	24	Plans for seeking research ethics committee/institutional review board (REC/IRB) approval	12
Protocol amendments	25	Plans for communicating important protocol modifications (eg, changes to eligibility criteria, outcomes, analyses) to relevant parties (eg, investigators, REC/IRBs, trial participants, trial registries, journals, regulators)	N/A
Consent or assent	26a	Who will obtain informed consent or assent from potential trial participants or authorised surrogates, and how (see Item 32)	5

For peer review only

	26b	Additional consent provisions for collection and use of participant data and biological specimens in ancillary studies, if applicable	N/A
Confidentiality	27	How personal information about potential and enrolled participants will be collected, shared, and maintained in order to protect confidentiality before, during, and after the trial	13
Declaration of interests	28	Financial and other competing interests for principal investigators for the overall trial and each study site	18
Access to data	29	Statement of who will have access to the final trial dataset, and disclosure of contractual agreements that limit such access for investigators	18
Ancillary and post-trial care	30	Provisions, if any, for ancillary and post-trial care, and for compensation to those who suffer harm from trial participation	N/A
Dissemination policy	31a	Plans for investigators and sponsor to communicate trial results to participants, healthcare professionals, the public, and other relevant groups (eg, via publication, reporting in results databases, or other data sharing arrangements), including any publication restrictions	15
	31b	Authorship eligibility guidelines and any intended use of professional writers	N/A
	31c	Plans, if any, for granting public access to the full protocol, participant-level dataset, and statistical code	N/A
**Appendices**			
Informed consent materials	32	Model consent form and other related documentation given to participants and authorised surrogates	N/A
Biological specimens	33	Plans for collection, laboratory evaluation, and storage of biological specimens for genetic or molecular analysis in the current trial and for future use in ancillary studies, if applicable	N/A

*It is strongly recommended that this checklist be read in conjunction with the SPIRIT 2013 Explanation & Elaboration for important clarification on the items. Amendments to the protocol should be tracked and dated. The SPIRIT checklist is copyrighted by the SPIRIT Group under the Creative Commons "Attribution-NonCommercial-NoDerivs 3.0 Unported" license.

For peer review only

BMJ Open

Evaluating the impact of a common elements based intervention to improve maternal psychological wellbeing and mother-infant interaction in rural Pakistan: Study protocol for a randomized controlled trial

Journal:	*BMJ Open*
Manuscript ID	bmjopen-2020-047609.R2
Article Type:	Protocol
Date Submitted by the Author:	20-Jun-2021

Complete List of Authors:	Huma, Zill-e-; Human Development Research Foundation, Implementation Science; University of Liverpool Faculty of Health and Life Sciences, Department of Primary Care and Mental Health<br>Gillani, Ayella; Human Development Research Foundation, Implementation Science<br>Shafique, Fakhira; Human Development Research Foundation, Implementation Science<br>Rashid, Alina; Human Development Research Foundation, Implementation Science<br>Mahjabeen, Bushra; Human Development Research Foundation, Implementation Science<br>Javed, Hashim; Human Development Research Foundation, Implementation Science<br>Wang, Duolao; Liverpool School of Tropical Medicine, Department of Clinical Sciences<br>Rahman, Atif; University of Liverpool Faculty of Health and Life Sciences, Department of Primary Care and Mental Health<br>Hamdani, Syed Usman; University of Liverpool Faculty of Health and Life Sciences, Department of Primary Care and Mental Health ; Human Development Research Foundation, Implementation Science

**Primary Subject Heading**:	Mental health
Secondary Subject Heading:	Mental health, Evidence based practice
Keywords:	MENTAL HEALTH, Community child health < PAEDIATRICS, Depression & mood disorders < PSYCHIATRY, Public health < INFECTIOUS DISEASES, Developmental neurology & neurodisability < PAEDIATRICS, Anxiety disorders < PSYCHIATRY

SCHOLARONE™
Manuscripts

I, the Submitting Author has the right to grant and does grant on behalf of all authors of the Work (as defined in the below author licence), an exclusive licence and/or a non-exclusive licence for contributions from authors who are: i) UK Crown employees; ii) where BMJ has agreed a CC-BY licence shall apply, and/or iii) in accordance with the terms applicable for US Federal Government officers or employees acting as part of their official duties; on a worldwide, perpetual, irrevocable, royalty-free basis to BMJ Publishing Group Ltd ("BMJ") its licensees and where the relevant Journal is co-owned by BMJ to the co-owners of the Journal, to publish the Work in this journal and any other BMJ products and to exploit all rights, as set out in our licence.

The Submitting Author accepts and understands that any supply made under these terms is made by BMJ to the Submitting Author unless you are acting as an employee on behalf of your employer or a postgraduate student of an affiliated institution which is paying any applicable article publishing charge ("APC") for Open Access articles. Where the Submitting Author wishes to make the Work available on an Open Access basis (and intends to pay the relevant APC), the terms of reuse of such Open Access shall be governed by a Creative Commons licence – details of these licences and which Creative Commons licence will apply to this Work are set out in our licence referred to above.

Other than as permitted in any relevant BMJ Author's Self Archiving Policies, I confirm this Work has not been accepted for publication elsewhere, is not being considered for publication elsewhere and does not duplicate material already published. I confirm all authors consent to publication of this Work and authorise the granting of this licence.

For peer review only

Evaluating the impact of a common elements based intervention to improve maternal psychological wellbeing and mother-infant interaction in rural Pakistan: Study protocol for a randomized controlled trial

Zill-e-Huma [1,2,3], Ayella Gillani [1,3], Fakhira Shafique [1,3], Alina Rashid [1,3], Bushra Mahjabeen [1,3] Hashim Javed[1,3], Duolao Wang [4], Atif Rahman [2], Syed Usman Hamdani [1,2,3]

[1]Human Development Research Foundation, Rawalpindi, Pakistan

[2]Department of Primary Care and Mental Health, University of Liverpool, Liverpool, United Kingdom

[3]Global Institute of Human Development, Shifa Tameer-e-Millat University, Islamabad, Pakistan

[4]Liverpool School of Tropical Medicine, Liverpool, United Kingdom

Corresponding Author:
Zill-e-Huma
Human Development Research Foundation (HDRF), Pakistan
E: zille.huma@hdrfoundation.org

Abstract

For peer review only

For peer review only - http://bmjopen.bmj.com/site/about/guidelines.xhtml

Introduction Millions of children in low resource settings are at high risk of poor development due to factors such as under nutrition, inadequate stimulation and maternal depression. Evidence-based interventions to address these risk factors exist, but often as a separate and overlapping package. The current study aims to evaluate the effectiveness of a common elements-based intervention to improve mother-infant interaction at 12-months post-partum.

Method and analysis A two-arm, single-blinded, individual randomized controlled trial (RCT) is being carried out in the community settings of the rural sub-district of Gujar Khan in Rawalpindi, Pakistan. 250 pregnant women in third trimester with distress (Self-Reporting Questionnaire (SRQ), cut-off score >9) have been randomized on 1:1 allocation ratio into intervention (n=125) and Treatment as Usual (TAU) arms (n=125). The participants in the intervention arm will receive 15 individual sessions of intervention on a monthly basis by non-specialist facilitators. The intervention involves components of early stimulation, learning through play, responsive feeding, guided discovery using pictures, behavioural activation and problem solving. The primary outcome is caregiver-infant interaction at 12-months post-partum. The secondary outcomes include maternal psychological wellbeing, quality of life, social support and empowerment. Infant secondary outcomes include growth, nutrition and development. The data will be collected at baseline, 6- and 12-months' post-partum. A qualitative process evaluation will be conducted to inform the feasibility of intervention delivery.

Ethics Ethics approval for the present study was obtained from the Human Development Research Foundation (HDRF) Institutional Review Board, Islamabad Pakistan.

Dissemination If proven effective, the study will contribute to scale-up care for maternal and child mental health in low resource settings, globally. The findings of the present study will be published in peer-reviewed journals and presented at conferences and community forums.

Trial registration: Registered prospectively with ClinicalTrials.gov as "a common elements-based intervention to improve maternal psychological well-being and mother-infant interaction". Identifier: NCT04252807. Study start and end date: September 2019 to March, 2021 (19-months).

Keywords: Maternal distress; mother-infant interaction; common elements treatment approach; nurturing care; low resource settings

Article summary

- To address the burden associated with poor infant development and perinatal distress we have developed a common elements based early intervention. The intervention is designed to be delivered through non-specialist facilitators in low resource community settings.
- In a single-blinded, individual randomized controlled trial, we will evaluate the impact of intervention using a pragmatic primary outcome measure i.e. 5-minute observation of a live caregiver-child interaction.
- The COVID-19 pandemic has resulted in limited face to face contact with the study participants and the intervention is being delivered via telephone or in-person sessions

For peer review only - http://bmjopen.bmj.com/site/about/guidelines.xhtml

where possible. We shall evaluate the feasibility and acceptability of blended intervention delivery methodology using mixed methods process evaluation.

- Continued participation of mother-infant dyads through the completion of the intervention and to the final assessment point at 12-months postpartum could be a challenge in the COVID-19 pandemic.

Introduction

In South Asia, 35-45% children are undernourished [1] and over 40 million lag behind in their cognitive and socio-emotional development [2]. The situation is compounded by maternal depression, which affects over a quarter of all women [3]. Suboptimal functioning and agency in depressed mothers is a barrier to implementation of any intervention directed at the young child [4]. Together, these factors have a negative impact on provision of nurturing care that is essential for optimal development [5-8].

Pakistan has a population of about 200 million people, of which three quarters live in rural areas. 47% of children under 5 years of age suffer from stunted growth and development in Pakistan [9]. By conservative estimate about eight million children do not achieve their full cognitive potential [2]. Prevalence studies in the proposed study area have reported high rates of perinatal depression in mothers ranging from 25-37% and a strong and independent association of maternal depression with low birth weight and under nutrition [10]. Studies have shown that antenatal stress can have detrimental effects on fetal development [11]. Lack of early interventions mean that many of these children might develop cognitive, social and emotional difficulties, contributing further to emotional and financial stress in the families [12]. Especially in the context of on-going COVID-19 pandemic, which is indirectly impacting maternal and child health outcomes due to disruption of health care services and lack of access to existing maternal and child health care during lockdown, delivering early interventions in community settings becomes imperative [13].

Considerable effort has gone into child survival programmes, leading to a reduction in child mortality rates over the last decade. However, improved child survival means there are many more children who are at-risk of developmental delay. Evidence-based interventions to address risk factors for poor child development exist, but often as separate and overlapping packages of interventions.

To address these challenges, we have developed a package of intervention using a common elements approach [14] that combines evidence-based elements from packages of care addressing early infant stimulation, responsive feeding and maternal distress [15-17]. These approaches recognize the importance of early critical years and role of the primary caregiver – normally the mother to provide nutrition and an optimal environment for interaction and stimulation. CEA is the concept of identifying components that overlap across multiple evidence-based practices for a specific problem area [14]. CEA combines a number of overlapping theoretical approaches into a single intervention with synergistic effects across the range of domains. The mother, in turn, receives psychosocial support, education and skills training specific to Early Child Development (ECD), provided via a well monitored and supervised delivery system.

We aim to evaluate the effectiveness of common elements-based intervention compared to usual care to improve mother-infant interaction along with improvements in maternal psychological wellbeing, infant growth, nutrition and development at 12-months post-partum in low resource rural community settings of Pakistan.

Hypotheses

For peer review only - http://bmjopen.bmj.com/site/about/guidelines.xhtml

We hypothesize that common element based integrated intervention will result in improving caregiver-child interaction measured using Observation of Mother and Child Interaction (OMCI) in intervention arm compared with the Treatment –As-Usual in control arm, at 12 months' postpartum. Our secondary hypotheses (exploratory) are that our intervention will result in improvement in infant growth, nutrition and development and will improve maternal psychological wellbeing and quality of life.

Methods and analysis

Study design

A two-arm, single-blinded, individual randomized controlled trial with mixed-methods process monitoring and evaluation is being carried out to evaluate the effectiveness of intervention. The participants are recruited from a rural sub-district, randomized into intervention and control arms, on a 1:1 allocation ratio.

Patient and public involvement

The multicomponent intervention is based on existing community programmes developed by the research team working collaboratively with the people from local communities including mothers with post-natal depression, fathers, extended family members, community health workers and local volunteers [4, 6, 12, 18]. The relevant stakeholders (community representatives and gate keepers and mental health experts) were involved through consultative workshops (Theory of Change workshops) in the formative research to develop a hypothesized pathway for the implementation of intervention in the study sub-district. The findings of the present study will be disseminated to participants through presentation at community forums.

Study settings

The present study is being conducted in sub-district of Gujar Khan in Rawalpindi Pakistan. The sub-district represents a typical rural area of Pakistan and have poor demographic and health profile indicators (with high fertility rate – 4 births per woman and low female literacy rate i.e. 45%) [19], where 1 in every 4 women suffer from perinatal depression [19, 20]. The infant mortality rate in Rawalpindi has been estimated to be 55 per thousand live births, the under-5 mortality rate is 82 per thousand live births and the percentage of underweight children below 5 years of age is 25% [21]. Primary health care is delivered through a network of Basic Health Units (BHUs), each providing care to about 15,000–20,000 people. Each BHU is staffed by a doctor, midwife, vaccinator, and 15–20 village-based Community Health Workers (CHWs) also called Lady Health Workers (LHWs). These women have completed secondary school and are trained to provide preventive maternal and child health care, and education in the community. Each LHW is responsible for about 100 households in her village. The participants shall be recruited from the catchment areas of LHWs. The study participants are permanent residents in the study sub-district and have been living here for past many generations. The implementing research organization has been working closely with the health department in the study area for last 15 years and has developed a trustable relationship with the community members. The study participants are being approached through Lady Health Workers (LHWs), who have trusting relationship with community, this makes the enrolment process and retention in the study more feasible. Also, convenience of participants is taken into account when assessment and intervention delivery sessions are scheduled. These factors are expected to improve the retention rate of participants in study over a longer period of time.

Research participants:

For peer review only - http://bmjopen.bmj.com/site/about/guidelines.xhtml

The target population for the present study is pregnant women in third trimester with distress (screen positive on Self-Reporting Questionnaire, cut-off score ≥ 9).

Eligibility criteria of participants

Inclusion criteria:

- Pregnant with third trimester (28 gestational week)
- Age 18-40 years
- Intend to reside in the study areas until the completion of the study
- Score ≥9 on the SRQ

Exclusion criteria

- Women who require immediate or on-going medical or psychiatric care reported.
- Severe previous or current obstetric morbidity including eclampsia and antepartum hemorrhage;
- Medical disorders that require inpatient management (e.g., diabetes, hypertension, thromboembolism, cardiac disease)

Sample size calculations

The power calculations are based on one primary outcome, i.e. mother-child interaction on Observation of Mother and Child Interaction (OMCI) tool [22] at 12 months' post-partum. The scoring of tool is based on the frequency of the occurrence of responsive behaviours of mother with higher scores indicating more responsive interactions. The effect size for the present study was estimated based on the findings of a similar study conducted in Pakistan, where OMCI was used as one of the outcomes to evaluate the effect of responsive stimulation intervention on caregiver-child interaction at 12 and 24-months post-partum [8]. The findings of the study showed mother–child dyads receiving responsive caregiving intervention had significantly higher mean scores on OMCI at 12 months (mean 32.3 [SD 8.3] vs mean 27.1 [SD 8.2], p <0.0001) than those who were allocated to control arm. Moreover, repeated-measures analysis showed that effects were sustained at 24-months. As the effect sizes for OMCI in Yousafzai et al 2015's study ranged between 0.6 to 0.9; therefore, a conservative effect size of 0.4 (two-sides hypothesis) at 12-months post-partum was proposed in the present study. The effect size was calculated using the two-sided t-test. Assuming an effect size of 0.4, with 80% power, 0.05 significance, and a two-sided hypothesis test and accounting for 20% attrition, we will need 250 caregiver-child dyads (125 in each arm).

Recruitment procedure

Before recruiting participants in the trial, the local community stakeholders were taken on-board. As mentioned in the study settings, the current study is being conducted in the rural sub-district of Gujar Khan, Rawalpindi district, Pakistan. The study sub-district consists of 36 Union Councils (UCs)- the smallest administrative units of the sub-district. Each UC has a Basic Health Unit (BHU) that provides primary health care to about 15,000–20,000 local population. Each BHU is staffed by a doctor, midwife, vaccinator, and 15–20 village-based Community Health Workers (CHWs) also called Lady Health Workers (LHWs). These women have completed secondary school and are trained to provide preventive maternal and child health care, and education in the community. Each LHW is responsible for about 100-150 households in her village and keeps a register of every new pregnancy in her catchment area. The research participants in the present

For peer review only - http://bmjopen.bmj.com/site/about/guidelines.xhtml

study is being recruited from the catchment areas of LHWs, where, every woman in their third trimester of pregnancy is being assessed for eligibility. Pregnant women in their third trimester (N=250), who fulfil the eligibility criteria, are being enrolled in the trial and individualized randomized into intervention and TAU arm (1:1 ratio). (Fig 1). Written informed consent is obtained from the study participants by trained assessment team. A complete record of assessment against eligibility criteria, and reasons for refusal is maintained for those who do not consent for participation in the trial.

All potential eligible pregnant women in third trimester are screened for psychological distress by research team using Self-Reporting Questionnaire (SRQ). Psychological distress is represented by subscales of depression/anxiety, somatic symptoms, reduced vital energy, and depressive thoughts during the past month. The scale reflects multidimensional nature of mental illnesses. It has 20 items scored on 0 or 1. A score of 1 indicates that the symptom was present during the past month; a score of 0 indicates that the symptom was absent. The maximum score is therefore 20, with a higher score indicating higher psychological distress. A score of ≥ 9 indicates increased risk of postnatal depression [8] and mothers with these scores have been recruited in the study.

Intervention

The intervention called 'Nurturing Care' combines common elements from evidence-based interventions for maternal psychosocial distress (e.g. WHO Thinking Healthy Program [17]), early stimulation and responsive feeding (e.g. UNICEF Care for Child Development Package [15]), and child care (e.g. WHO Caregivers Skills Training program [16]) for optimal development of at risk mother and children.

The intervention package aims to improve a) mother psychological distress by promoting problem solving, developing empathic listening, improving family support, using guided discovery through pictures and behavioural activation; b) child development through psycho-education, responsive breastfeeding and c) mother-infant interaction through early stimulation and learning through play (see Table 1 for description of intervention sessions).

The intervention is designed to be delivered by non-specialist facilitators in 15 monthly sessions in community settings and commences from third trimester of pregnancy and ends at 12-months postpartum. During the COVID-19 pandemic, when the in-person sessions are not possible, we are delivering the scheduled intervention session via telephone. All those sessions delivered via telephone are not repeated in-person.

Table 1: Description of intervention sessions

Sessions	Content	Timing	Recipients
1: Introduction and Rapport building	- Introducing program and engaging mother and her family<br>- Developing empathetic relationship with mother<br>- Ensuring family support system	Beginning of 7th month of pregnancy	Mother and family members
2: Maternal wellbeing	- Assessment of psychosocial problems of mother	7th month of pregnancy	Mother and family members

For peer review only - http://bmjopen.bmj.com/site/about/guidelines.xhtml

	- Use of guided discovery and behavioral activation for improving mother's health (strategies for improving overall well-being, including diet, rest, relaxation and sleep)		
3: Improving mother relationship with significant others	- Thought challenging and behavior activation using guided discovery (strategies for improving interpersonal relationships and mobilizing social support)<br>- Awareness about complication during pregnancy	8th month of pregnancy	Mother and family members
4: Improving mother relationship with infant during pregnancy	- Thought challenging and behavior activation using guided discovery (strategies for improving bond with the infant during pregnancy. knowledge and planning of safe delivery and importance of colostrum)	9th month of pregnancy	Mother
4: Follow-up session after delivery	- Following up after child birth<br>- Psycho-education about child development	Immediate post-delivery	Mother
5: Improving child's physical health	- Thought challenging and behavior activation using guided discovery (strategies for awareness on benefits of exclusive breastfeeding)<br>- Responsive breast feeding<br>- Addressing the concept of child crying because of insufficient milk<br>- Increasing breast milk by diet and improved frequency- addressing insufficiency of milk<br>- Family support for breast feeding<br>- Assessment of post-partum psychosocial problems of mother	8 weeks postnatal	Mother
5A: Improving maternal health	- Assessment of psychosocial problems of mother	9 weeks postnatal	Mother

For peer review only - http://bmjopen.bmj.com/site/about/guidelines.xhtml

	- Use of guided discovery and behavioral activation for improving mother's health		
5B: Improving maternal health	- Use of guided discovery and behavioral activation for improving mother's relationship with others	10 weeks postnatal	Mother
5C: Improving maternal health	- Use of guided discovery and behavioral activation for improving mother's relationship with child	11 weeks postnatal	Mother
6: Importance of mother child interaction	- Exclusive Breast Feeding (EBF) for 6months<br>- Birth spacing<br>- Suitable environment- birth spacing<br>- Interaction, love and care for child<br>- Selecting play activity and conducting with mother and child	12 weeks postnatal	Mother and infant
7: Follow up on early stimulation activities	- Revision of activities discussed in session 6<br>- Importance of vaccination	16 weeks postnatal	Mother and Child
8: Awareness on birth spacing; improving child's health	Thought challenging and behavior activation using guided discovery (strategies for awareness on birth spacing and its relation to child's health)<br><br>- Exclusive breast feeding for 6-month<br>- Birth spacing<br>- Suitable environment- birth spacing<br>- Modern methods of contraception<br>- Interaction, love and care for child<br><br>Selecting play activity and conducting with mother and child	20 weeks postnatal	Mother

For peer review only - http://bmjopen.bmj.com/site/about/guidelines.xhtml

9: Introduction to solid food	- Interaction and play with child<br>- Selecting play activity and conducting with mother and child<br>- Introduction to Solid food and concept of super food<br>- Food and hygiene<br>- Continuation of breastfeeding with weaning at 6m<br>- Importance of vaccination	24 weeks postnatal	Mother
10: Introduction to mother child interaction. Improving mother child interaction through responsiveness and praise	- Skill based activities to promote mother-child interaction<br>- Homework charts are given to monitor the activity.<br>- Learning through play activities are reinforced<br>-	28 weeks postnatal	Mother and infant
11: Importance of following child's interest	- Skill based activities to promote mother-child interaction<br>- Homework charts are given to monitor the activity.<br>- Learning through play activities are reinforced	32 weeks postnatal	Mother and infant
12: Importance of following child's lead	- Skill based activities to promote mother-child interaction<br>- Homework charts are given to monitor the activity.<br>- Learning through play activities are reinforced	36 weeks postnatal	Mother and infant
13: Promote child language development	- Skill based activities to promote mother-child interaction<br>- Homework charts are given to monitor the activity.<br>- Learning through play activities are reinforced	40 weeks postnatal	Mother and infant
14: Scaffolding	- Skill based activities to promote mother-child interaction<br>- Homework charts are given to monitor the activity.<br>- Learning through play activities are reinforced	44 weeks postnatal	Mother and infant

For peer review only - http://bmjopen.bmj.com/site/about/guidelines.xhtml

15: Review of all 5 strategies	Revision of all strategies and next line of action.<br>Learning through play activities are reinforced	48 weeks postnatal	Mother and infant

Training and supervision of non-specialist facilitators in 'Nurturing Care' intervention

The delivery agents of the 'Nurturing Care' intervention are female non-specialist facilitators who are graduates in psychology/social sciences with little or no prior experience of delivering psychosocial interventions to mothers with perinatal distress and their new-born infants and have achieved competency to deliver 'Nurturing Care' intervention following training and practice sessions. These non-specialist facilitators were selected from the universities in the study sub-district through a job advertisement and interviews.

A cascade model of training and supervision has been followed to deliver 'Nurturing Care' intervention. The training consisted of classroom teaching of basic theoretical concepts of 'Nurturing Care' intervention, its strategies, group discussions and role-plays. The program trainers trained 15 non-specialist facilitators. The training consisted of 10-days class-room training followed by practice cases under the supervision of program trainer. The participants of this practice group were pregnant women without distress and their infants, selected from the same community after seeking informed consent for participation in the training. The program trainer performed live competency rating during practice cases using an adapted version of ENhancing Assessment of Common Therapeutic factors (ENACT) tool for 'Nurturing Care' intervention (Kohrt et al. 2015). Only competent non-specialist facilitators (mean score above 2.5 on all domains of adapted ENACT) have been allowed to deliver 'Nurturing Care' intervention. Each non-specialist facilitator has been assigned a case load of 8-10 mothers to deliver intervention. The fidelity of the intervention is being evaluated using an adapted ENACT tool developed for the program. 20% of randomly selected intervention sessions from each non-specialist facilitators at different stages of the intervention delivery will be rated by the program trainers to assess the fidelity of intervention delivery by non-specialist facilitators.

Supervision

Program trainers are experts in perinatal mental health and infant development and provide weekly supervision to non-specialist facilitators to ensure intervention fidelity. Supervision involves discussion of difficulties encountered in delivering intervention delivery and, as well as self-care of the non-specialist facilitators.

Treatment as Usual (TAU)

As a part of treatment-as-usual, participants in both study arms receive routine visits by the Lady Health Workers (LHWs) of their respective areas. The LHWs are trained to provide preventive maternal and child care including psycho-education about family planning and maternal and child health; provide free of cost contraceptives and antenatal-iron-folic acid supplements throughout the pregnancy starting from second trimester via door to door visits to the households of their allocated areas. LHWs visit new mothers on a monthly basis to monitor the progress of a newborn baby on growth parameters such as weight and height. Moreover, they are given additional duties (provide information about infectious disease or to administer oral polio drops to children) which requires regular visits to houses. In coordination with traditional birth attendants and affiliated nearest health facility, LHWs also provide referral service to mothers for safe motherhood. In the current study, the number of visits mothers receive by LHWs will be the same for each trial participant regardless of their allocation status.

For peer review only - http://bmjopen.bmj.com/site/about/guidelines.xhtml

If, during the study's assessments time-points participants in both arms show severe psychiatric disorders that require immediate specialist treatment and follow-up, they will be referred to tertiary mental health care facility, Institute of Psychiatry, Benazir Bhutto Hospital, Rawalpindi. A complete record of services received by trial participants in both arms will be made using the Client Services Receipt Inventory (CSRI).

Outcomes measures

The effectiveness of intervention will be evaluated on a range of caregiver and child outcomes. The outcome measures will be administered by the trained assessment team blind to the allocation status of participants at baseline (at the time of recruitment i.e. 3rd trimester of pregnancy), 6 and 12-months' postpartum. The self-report outcome measures will be orally administered by the trained assessment team blind to the allocation status of the participants. The detail of the outcome measures is given below (see Table 2 for schedule of assessments);

Primary Outcome

Caregivers Child Interaction (CCI): Maternal responsive caregiving behaviours will be assessed at 12-months' postpartum across two-arms (primary endpoint) using the **Observation of Mother and Child Interaction (OMCI) tool [22].** OMCI tool is based on responsive parenting framework proposed by Landry and colleagues [23]. The tool comprises of 19 items related to maternal and child behaviors. Maternal behaviors include maternal affect, maternal touch, maternal verbalization, sensitivity and contingent responses, scaffolding, language stimulation, and focus; whereas, child outcomes include, child affect, child focus, child's communication efforts, and mutual enjoyment. The scoring is done by recording the frequency of the occurrence of each of the above mentioned behaviors during the mother-child interaction. The sum is calculated by adding the scores of mother and child domains; where higher score indicates more responsive interactions. The tool has been designed to live rate mother-child interaction of 2-years old toddlers in low resource settings of rural Pakistan. It has been extensively used in similar population in Pakistan and found to be sensitive to detect change in mother-child interaction as a result of receiving responsive caregivers intervention at 12 and 24-months post-partum [22]. In the present study, the research assistants will observe a live 5-minute mother-infant interaction while mother and infant will play together with a picture book.

Infant secondary outcomes

1. **Bayley's Scales of Infant Development (BSID III**) [24] will be used to measure cognitive, language, motor, socio-emotional development and adaptive behaviour at 12-months' postpartum. The tool has been used previously in similar South Asian settings [25, 26].
2. **Ages and Stages Questionnaire 3rd Edition (ASQ3) [27]** will be used to measure developmental delays in communication, gross motor, fine motor, problem solving, and personal social domains at 12-months' postpartum. It provides cut-off score in 5 domains of development that indicates possible need for further evaluation. It highlights results that fall in a "monitoring zone," to make it easier to keep track of children at-risk. The tool has been validated to use in South Asian community settings [26, 28].
3. Home environment measured by **Home inventory [29]:** Quality and quantity of stimulation and support in the child's home will be measured using home inventory at 12-months' postpartum. The tool has been used validated to use in Pakistan [30].

For peer review only - http://bmjopen.bmj.com/site/about/guidelines.xhtml

4. **Anthropometric measurements [31]**: Infants in both groups will be weighed and measured with standard techniques at 12-months' postpartum. Growth data will be converted into SDs (Z scores) for weight and length with WHO Anthro (version 3.2.2).
5. **Early breastfeeding (EBF) [32]**: Assessors will document what the infant was fed in the last 24 hours at 6 months postpartum. This information will be categorized as either EBF, partial breastfeeding (giving an infant some breastfeeding, and some additional foods, either milk, cereal, or other food items), or no breastfeeding. If the infant will not exclusively breastfeed, details of when (age of infant) and why EBF was discontinued will be recorded. At the first 6-months follow-up, details about early initiation, discarding colostrum, use of pre-lacteals, and reasons for delaying or not initiating breastfeeding will be assessed.
6. The number of **diarrhoeal episodes** in the infants during the 2 weeks before interview will be recorded at 6 and 12-months' postpartum, with a questionnaire used in previous studies [33]. Diarrhoea will be defined as three or more unformed stools passed in 24 hours, and a diarrhoeal episode will be defined as being separated from another episode by at least 3 diarrhoea-free days.
7. Records of **immunisation** will be assessed for all infants in the study, and infants will be classified as those with or without up-to-date immunisation status 6 and 12-months' postpartum.

Maternal secondary outcomes;

8. Maternal distress will be measured by **the Self Reporting Questionnaire (SRQ**) [34] at baseline, 6 and 12-months postpartum. SRQ is a 20-item self-report measure to detect non-specific psychological distress, developed by the World Health Organization (WHO). Psychological distress is represented by subscales of depression/anxiety, somatic symptoms, reduced vital energy and depressive thoughts. The SRQ items are scored 0 or 1. A score of 1 indicates the presence of symptoms of psychological distress during past month and a score of 0 indicates absence of symptoms. The maximum score indicates presence of higher psychological distress. A score of > 9 indicates increased risk of maternal distress/post-partum depression [8, 35].
9. Caregivers' health related quality of life will be measured by **WHO Quality of Life (WHOQoL)- Brief version** at baseline and 12-months postpartum. WHOQoL assess the participants position in life in the context of the culture and value systems in which they live and in relation to their goals, expectations, standards and concerns. WHOQoL comprises of 4 domains namely physical health, psychological health, social relationships and environment. Items are rated on a 5-point Likert scale, where 1 represents "disagree" or "not at all" and 5 represents "completely agree" or "extremely".
10. The level of social support will be measured using **Multi-Dimensional Scale of Perceived Social Support (MSPSS) [36]** will be measured baseline and 12-months' postpartum. It includes 12 items which cover three dimensions: support from family, friends and significant other. Each item is rated on a 7-point Likert-scale (1 = *very strongly disagree*; 7 = *very strongly agree*). A total score is calculated by summing the responses of all items (range 12–84) with higher scores indicating higher levels of perceived social support.
11. Caregiver's level of functioning will be measured using **World Health Organization Disability Assessment Schedule (WHODAS) [37]** at baseline and 12-months' post-partum. WHODAS assesses participants' health-related difficulties in the level of functioning in six domains of life (understanding and communicating; moving and getting around; attending to one's hygiene, dressing, eating and staying alone; interacting with other people; domestic

For peer review only - http://bmjopen.bmj.com/site/about/guidelines.xhtml

responsibilities, leisure, work and school and joining in community activities, participating in society) over the past 30 days.

12. **Maternal Financial empowerment**: Women's empowerment will be measured at baseline and 12-months postpartum, using a questionnaire consisting of: (a) two items previously used in the same population [38] that assess financial empowerment (i.e., whether a woman is given a lump-sum of money for day-to-day expenses and whether she can make independent decisions about its use). Women who answer '*yes*' to both items on financial empowerment will be classified as 'empowered'.
13. Utilization of health care will be measured by **Client Service Receipt Inventory (CSRI)** adapted for perinatal population of Pakistan, at 12 months postpartum [32].

Table 2: Schedule of assessments

Sr. no.	Outcomes		Time points		
		Screening	Baseline 3rd trimester of pregnancy	6-months post-partum	12 months postpartum
Primary outcome					
1.	OMCI				X
Secondary outcomes					
2.	SRQ	X	X	X	X
3.	WHO-QoL		X		X
4.	MSPSS		X		X
5.	WHODAS		X		X
6.	Maternal empowerment-brief questionnaire		X		X
7.	BSID-III				X
8.	ASQ3				X
9.	Home inventory				X
10.	Weight and height measurements				X
11.	Early breastfeeding-brief questionnaire			X	
12.	Diarrhoeal episodes -brief questionnaire			X	X
13.	Immunization-record form			X	X
14.	CSRI				X

OMCI, Observation of Mother-Child Interaction; SRQ, Self-reporting Questionnaire; WHO-QoL, World Health Organization, Quality of Life; MSPSS, Multi-Dimensional Scale of Perceived Social Support; WHODAS, WHO Disability Assessment Schedule; BSID-III, Brief questionnaire, Bayley Scale of Infant Development-III; ASQ3, Ages and Stages Questionnaire-3; CSRI, Client Service Receipt Inventory

As the study is being conducted amidst COVID-19, all in-person assessments will be conducted by following appropriate precautionary measures.

For peer review only - http://bmjopen.bmj.com/site/about/guidelines.xhtml

Randomization and blinding

The participants were randomized into intervention or control arm on a 1:1 basis using computerized software by an independent researcher. The allocation concealment was ensured by keeping random sequence in sequentially numbered, opaque, sealed envelopes, at the off-site centre. Only assessment team (conducting baseline and follow-up assessment) will be blind to the treatment allocation of trial participants. To maintain the blinding, all trial participants will be briefed, prior to the assessment about the significance of blinding and the importance of not disclosing their allocation status to the assessment team members. If un-blinding does occur, this will be documented, the assessment will be halted and a new assessor will be assigned to complete the assessment.

Safety measures

In the present study, a referral pathway, based on the existing referral system, has been established to ensure the safety of the study participants. The Institute of Psychiatry (IoP) is the tertiary mental health care facility in the north of Pakistan and situated within the public tertiary healthcare facility and teaching hospital, the Benazir Bhutto Hospital, Rawalpindi. It caters to the mental health needs of the populations in the metropolitan city of Rawalpindi and is the specialist referral facility for common mental health problems identified at the adjacent primary healthcare centers. In our study, if a participant discloses any event of abuse, intimate partner violence, or is at imminent risk of harm to herself and others, identified at the time of assessment or during the intervention delivery, the research team will record the event and referral to the IoP will be made for appropriate medical, medico-legal and psychosocial assessment and rehabilitation by a multi-disciplinary team led by qualified psychiatrists.

Adverse events reporting

All adverse events (AEs) and serious adverse events (SAEs) reported by the trial participants, their husband and in-laws of recruited participants, LHWs or observed by the investigator or the research team (assessment & intervention team) will be recorded by data manager. AE is defined as any undesirable experience occurring to a subject during the time frame of the study, whether or not considered relevant to the research procedure. All AEs will be collected by assessment team at 12-months' postpartum follows and will be reviewed periodically by trial steering committee which will determine the necessary steps to be taken with respect to the ongoing trial.

Data management

The baseline and follow-up assessments data will be collected by the assessment team on android based application using hand held devices (tablets). Data will be stored at the secure web server and will be checked for consistency & quality at the end of each day. All data sets will be stored in password protected computer, only accessible by the responsible individual. The data collected in hard copies (collected by the interviewers) will be safely stored in locked cabinets. Qualitative data will be fully anonymized and coded. No identifying names or details will be recorded.

Statistical analysis

The findings will be reported according to the CONSORT guidelines for RCT [39] (Figure 1). This will include the flow of research participants through each stage of the trial, including the number eligible, randomly assigned, receiving the intended treatment, completing the study protocol and analysed for the primary outcome. The primary analyses will be on intent-to-treat

For peer review only - http://bmjopen.bmj.com/site/about/guidelines.xhtml

basis consisting of all participants included according to the arms in which they are randomized and secondary analyses will be based on per-protocol population.

For the analysis of the primary outcome (caregiver-child interaction across two-arms at 12-months post-partum), a linear regression model will be employed with treatment as the sole predictor. In addition, adjusted linear model analysis will be performed with the pre-specified covariates (gender of infant, mother's education and severity of mothers' psychosocial distress measured using SRQ) measured at baseline being added into the above linear model. The crude and adjusted mean differences in the primary outcome together with its 95% confidence intervals at 12-months will be derived from the linear models. In addition, subgroup analysis of primary endpoint will be performed on the above pre-specified covariates.

Analysis of secondary continuous outcomes with single follow-up measurement will be done in a similar fashion as the primary endpoint analysis. Analysis of secondary continuous outcomes with repeated follow-up measurement will be performed using a linear mixed model with treatment visit, interaction between treatment and visit as fixed effects, the baseline value of the outcome as covariate, if it is available and subject as random effects. The analysis of binary outcomes will also use a generalized linear/mixed model depending on whether there will be a repeated measurement. Odds ratios with their 95% confidence intervals will be derived from the generalized linear/mixed model analysis. Missing primary outcome and secondary outcomes with a single measurement will not be imputed; whereas, missing secondary outcomes with repeated measurements will be imputed in sensitivity analysis using the last observation carried forward strategy. Missing baseline covariates will be imputed using simple imputation methods in the covariate adjusted analysis based on the covariate distributions in the sample. For a continuous variable, missing values will be imputed from random values from a normal distribution with mean and SD calculated from the available sample. For a categorical variable, missing values will be imputed from random values from a uniform distribution with probabilities P_1, P_2, …, and P_k from the sample. Seed for the imputation will be set as the date of data analysis. The study is powered to make a single comparison in the primary outcome at 12 months only. All analyses will be described in detail in the statistical analysis plan. SAS 9.4 and SPSS 21 will be employed for the statistical analyses.

Potential mediators

We will explore pathways through which the intervention may influence primary outcome (i.e. mother-child interaction). There is strong empirical evidence that the quality of the mother-child interactions, such as her sensitivity and responsiveness, is associated with both maternal depression and child development [40-43]. there is also some evidence that perinatal depression interventions, especially ones that include a parenting component, can improve maternal distress; maternal responsiveness, resulting in improved developmental outcomes [44]. We will assess maternal responsiveness with the maternal items of OMCI that measure maternal responsive behaviors during mother-child interaction. The OMCI has been used to evaluate the impact of maternal interventions on maternal responsiveness [45]. Maternal distress will be assessed using SRQ. The tool has been used extensively in the study settings previously to measure maternal distress [46].

Social support: Low social support is a consistent predictor of mental ill health among mothers [47, 48]. Social support can be theorized to be either a mediator or effect modifier; since building social support is a key feature of the integrated intervention, we hypothesize that increased social support will mediate the relationship between the intervention and child outcomes. *Maternal*

For peer review only - http://bmjopen.bmj.com/site/about/guidelines.xhtml

empowerment: Maternal empowerment will be assessed using two items previously used in the same population [38]. *Breastfeeding and other health related variables*; Perinatal depression interventions similar to this integrated intervention have resulted in higher rates of exclusive breastfeeding at 6 months and increased likelihood of completion of scheduled immunizations [6]. These health-related behaviours may contribute to the positive effect of intervention on improving maternal wellbeing and infant development, we will assess the impact of behaviours related to feeding (breastfeeding, introduction of solid food) and immunizations on mother-child interaction.

Cost-effectiveness analysis

Health economic analysis will be conducted to determine the difference in costs and outcomes in the intervention as compared to the control arm. The cost of the intervention will be calculated based on the sum of materials, personnel training, and delivery. We will then compare the combination of the cost of the intervention and the service cost data with mother-child interaction summary scores. If the intervention results in more favourable summary scores (improvement in mother-infant interaction) and lower total costs, it will be described as superior to no intervention option. If, in contrast, the intervention results in more favourable scores but is also more expensive, then the cost-effectiveness calculation will be based on the cost and scores of mother-infant interaction outcomes.

Primary analysis will be of total costs over the 12-months post-partum follow-up treatment period. Recognizing that cost data are often skewed, the bootstrap technique will be applied. The sampling with replacement from original observed pairs of costs and effects will be employed to maintain correlation structure between costs and benefits, and bootstrapping sampling will be repeated 1000 times. For each bootstrap sample, an estimate of differential total mean costs and expected mean effectiveness will be calculated. The 95% CIs for the differential estimates will be derived from the 25th and 97.5^{th} percentiles [49, 50].

Cost-effectiveness will be assessed by combing costs with the primary outcome measure (i.e. mother-infant interaction) scores in incremental cost-effectiveness analysis. Repeat resampling from the costs and effectiveness data (bootstrapping) will be used to calculate the probability that each of the treatment is the optimal choice, subject to a range of possible maximum values (ceiling ratio) that a decision-maker might be willing to pay for a unit improvement in mother-infant interaction scores. The results of the cost-effectiveness will be reported as incremental cost-effectiveness ratios, and acceptability curves which summarise the information contained in a cost-effectiveness plane [51].

Mixed-methods evaluation

We will evaluate the feasibility of our intervention program using mixed methods study design. Mixed-methods evaluation of implementation will be carried out to assess assumptions underlying the intervention strategy and will cover other aspects of program implementation such as number of research participants who signed-up and complete the intervention and satisfaction with intervention, difficulties and successes in carrying out intervention activities, acceptability, feasibility and appropriateness of using integrated intervention.

Satisfaction with the intervention and barriers and facilitators to implementation, feasibility of workload, and difficulties and successes in carrying out intervention activities and satisfaction with training and supervision will be explored through semi-structured interviews with the relevant stakeholders.

Ethics

For peer review only - http://bmjopen.bmj.com/site/about/guidelines.xhtml

Ethics approval for the present study was obtained from the Human Development Research Foundation (HDRF) Institutional Review Board, Islamabad Pakistan. Informed consent will be obtained from all participants prior to data collection. Privacy and confidentiality of the research participants will be ensured throughout the study. Trial management will be provided by Trial Steering Committee (TSC) that comprises Principal Investigator (PI), Co-Investigator (s), trial coordinator, senior researchers and intervention staff who will meet monthly.

Dissemination

There is a growing evidence base on the feasibility, acceptability and effectiveness of peer/parent delivered, psychosocial and early child development interventions [6, 18, 52]. Training and supervision of large numbers of health workers in new interventions is a key barrier to scale up of programs. Our intervention combines evidence-based elements into a single package, making delivery more effective and synergistic, through a single delivery agent i.e. non-specialist facilitator. The use of non-specialist facilitators in disseminating the interventions has the potential to extend the reach of the intervention in a cost-effective fashion. If proven effective, the study will contribute to scale-up care for maternal and child mental health in low resource settings globally. The Principal Investigator, trial coordinator and trial statistician will have access to the clean data sets. De-identified trial data can be accessed by contacting Principal Investigator upon reasonable request. We will disseminate our results to key stakeholders. Dissemination activities will include publication of study results in open access, peer-reviewed journals, and presentations at academic conferences and community forums.

References:

1. Black, R., et al., *Maternal and child undernutrition and overweight in low-income and middle-income countries.* The lancet, 2013. **382**(9890): p. 427-451.
2. Grantham-McGregor, S., et al., *Child Development in developing countries 1: Developmental potential in the first 5 years for children in developing countries.* Lancet, 2007(369): p. 60-70.
3. Fisher, J., et al., *Prevalence, nature, severity and correlates of postpartum depressive symptoms in Vietnam.* BJOG: An International Journal of Obstetrics & Gynaecology, 2004. **111**(12): p. 1353-1360.
4. Rahman, A., et al., *Impact of maternal depression on infant nutritional status and illness - A cohort study.* Archives of General Psychiatry, 2004. **61**(9): p. 946-952.
5. Hurley, K.M., A.K. Yousafzai, and F. Lopez-Boo, *Early child development and nutrition: a review of the benefits and challenges of implementing integrated interventions.* J Advances in nutrition, 2016. **7**(2): p. 357-363.
6. Rahman, A., et al., *Cognitive behaviour therapy-based intervention by community health workers for mothers with depression and their infants in rural Pakistan: a cluster-randomized controlled trial.* The Lancet, 2008. **372**(9642): p. 902-909.
7. Yousafzai, A.K., et al., *Effect of integrated responsive stimulation and nutrition interventions in the Lady Health Worker programme in Pakistan on child development, growth, and health outcomes: a cluster-randomized factorial effectiveness trial.* The Lancet, 2014. **384**(9950): p. 1282-1293.
8. Yousafzai, A.K., et al., *Effects of responsive stimulation and nutrition interventions on children's development and growth at age 4 years in a disadvantaged population in*

For peer review only - http://bmjopen.bmj.com/site/about/guidelines.xhtml

Pakistan: a longitudinal follow-up of a cluster-randomized factorial effectiveness trial. The Lancet Global Health, 2016. **4**(8): p. e548-e558.
9. Stevens, G.A., et al., *Trends in mild, moderate, and severe stunting and underweight, and progress towards MDG 1 in 141 developing countries: a systematic analysis of population representative data.* 2012. **380**(9844): p. 824-834.
10. Rahman, R. Harrington, and J. Bunn, *Can maternal depression increase infant risk of illness and growth impairment in developing countries?* Journal of Child Care, health and development 2002. **28**(1): p. 51-56.
11. Glover, V., *Maternal depression, anxiety and stress during pregnancy and child outcome; what needs to be done.* Best Pract Res Clin Obstet Gynaecol, 2014. **28**(1): p. 25-35.
12. Hamdani, S.U., et al., *Family networks to improve outcomes in children with intellectual and developmental disorders: a qualitative study.* J International journal of mental health systems, 2014. **8**(1): p. 7.
13. Busch-Hallen, J., et al., *Impact of COVID-19 on maternal and child health.* Lancet Glob Health, 2020. **8**(10): p. e1257.
14. Chorpita, B.F., E.L. Daleiden, and J.R. Weisz, *Identifying and selecting the common elements of evidence based interventions: A distillation and matching model.* Mental health services research, 2005. **7**(1): p. 5-20.
15. UNICEF. *Care for Child Development Package*. 2012; Available from: https://www.unicef.org/earlychildhood/index_68195.html.
16. Salomone, E., et al., *Development of the WHO Caregiver Skills Training Program for Developmental Disorders or Delays.* 2019. **10**(769).
17. WHO. *Thinking Healthy*. 2015 [cited 2020 Sep 16]; Available from: https://www.who.int/mental_health/maternal-child/thinking_healthy/en/.
18. Zafar, S., et al., *Integrating maternal psychosocial well-being into a child-development intervention: the five-pillars approach.* Ann N Y Acad Sci, 2014. **1308**: p. 107-17.
19. NIPS. *Pakistan demographic and health survey 2012–13.* 2013 [cited 2015 April 15]; Available from: https://dhsprogram.com/pubs/pdf/fr290/fr290.pdf.
20. Rahman, A. and F. Creed, *Outcome of prenatal depression and risk factors associated with persistence in the first postnatal year: Prospective study from Rawalpindi, Pakistan.* Journal of Affective Disorders, 2007. **100**(1-3): p. 115-121.
21. Bureau of Statistics and UNICEF Pakistan, *District based Multiple Indicator Cluster Survey (MICS), 2003-2004: Punjab*. 2003, Government of Punjab, Planning and Development Department, Federal Bureau of Statistics Islamabad United Nations Children's Fund, 2004: Lahore
22. Rasheed, M.A. and A.K. Yousafzai, *The development and reliability of an observational tool for assessing mother–child interactions in field studies-experience from Pakistan.* Child: care, health and development, 2015. **41**(6): p. 1161-1171.
23. Landry, S.H., et al., *Does early responsive parenting have a special importance for children's development or is consistency across early childhood necessary?* Developmental psychology, 2001. **37**(3): p. 387.
24. Albers, C.A. and A.J. Grieve, *Test review: Bayley, N.(2006). Bayley scales of infant and toddler development–third edition. San Antonio, TX: Harcourt assessment.* Journal of Psychoeducational Assessment, 2007. **25**(2): p. 180-190.

For peer review only - http://bmjopen.bmj.com/site/about/guidelines.xhtml

25. Kvestad, I., et al., *The assessment of developmental status using the Ages and Stages questionnaire-3 in nutritional research in north Indian young children.* Nutr J, 2013. **12**: p. 50.
26. Sikander, S., et al., *Cohort Profile: Perinatal depression and child socioemotional development ; the Bachpan cohort study from rural Pakistan.* BMJ Open, 2019. **9**(5): p. e025644.
27. Squires, J., D.D. Bricker, and E. Twombly, *Ages & stages questionnaires*. 2009: Paul H. Brookes Baltimore, MD.
28. Bhopal, S.S., et al., *Using the Mothers Object Relations Scale for early childhood development research in rural India: Findings from the Early Life Stress Sub-study of the SPRING Cluster Randomized Controlled Trial (SPRING-ELS).* Journal of Wellcome Open Research, 2021. **6**(54): p. 54.
29. Caldwell, B. and R. Bradley, *Home observation for measurement of the environment*. 1984: University of Arkansas at Little Rock Little Rock.
30. Yousafzai, A.K., et al., *Parenting Skills and Emotional Availability: An RCT.* Pediatrics, 2015. **135**(5): p. e1247-57.
31. WHO Expert Committee, *Physical status: the use and interpretation of anthropometry.* http://helid. digicollection. Org/en/d/Jh0211e/, 1995.
32. Sikander, S., et al., *Cognitive-behavioral counseling for exclusive breastfeeding in rural pediatrics: a cluster RCT.* Pediatrics, 2015: p. peds. 2014-1628.
33. Rahman, A., et al., *Maternal depression increases infant risk of diarrhoeal illness:–a cohort study.* Archives of Disease in Childhood, 2007. **92**(1): p. 24-28.
34. Beusenberg, M., J.H. Orley, and W.H. Organization, *A User's guide to the self reporting questionnaire (SRQ*. 1994, Geneva: World Health Organization.
35. Santos, I.S., et al., *Comparing validity of Edinburgh scale and SRQ20 in screening for post-partum depression.* Clinical practice and epidemiology in mental health : CP & EMH, 2007. **3**: p. 18-18.
36. Dahlem, N.W., G.D. Zimet, and R.R. Walker, *THE MULTIDIMENSIONAL SCALE OF PERCEIVED SOCIAL SUPPORT - A CONFIRMATION STUDY.* Journal of Clinical Psychology, 1991. **47**(6): p. 756-761.
37. Üstün, T.B., et al., *Developing the World Health Organization disability assessment schedule 2.0.* Bulletin of the World Health Organization, 2010. **88**: p. 815-823.
38. Rahman, A., et al., *Effective treatment of perinatal depression for women in debt and lacking financial empowerment in a low-income country.* The British Journal of Psychiatry, 2012. **201**(6): p. 451-457.
39. Moher, D., et al., *CONSORT 2010 Explanation and Elaboration: updated guidelines for reporting parallel group randomized trials.* 2010. **340**: p. c869.
40. Appelbaum, M., et al., *The effects of infant child care on infant-mother attachment security: Results of the NICHD study of early child care.* Child Development, 1997. **68**(5): p. 860-879.
41. Black, M., et al., *Depressive symptoms among rural Bangladeshi mothers: implications for infant development.* Journal of Child Psychology and Psychiatry, 2007. **48**(8): p. 764-772.
42. Schmid, B., et al., *Quality of early mother-child interaction associated with depressive psychopathology in the offspring: A prospective study from infancy to adulthood.* Journal of Psychiatric Research, 2011. **45**(10): p. 1387-1394.

For peer review only - http://bmjopen.bmj.com/site/about/guidelines.xhtml

43. Tamis-LeMonda, C.S., M.H. Bornstein, and L. Baumwell, *Maternal responsiveness and children's achievement of language milestones.* Child Development, 2001. **72**(3): p. 748-767.
44. van Doesum, K.T.M., et al., *A randomized controlled trial of a home-visiting intervention aimed at preventing relationship problems in depressed mothers and their infants.* Child Development, 2008. **79**(3): p. 547-561.
45. Beeber, L.S., et al., *Short-Term In-Home Intervention Reduces Depressive Symptoms in Early Head Start Latina Mothers of Infants and Toddlers.* Research in Nursing & Health, 2010. **33**(1): p. 60-76.
46. Rahman, A., *Screening for postnatal depression in the developing world: a comparison of the WHO Self-Reporting Questionnaire (SRQ-20) and the Edinburgh Postnatal Depression Screen (EPDS).* J Pak Psychiatr Soc, 2005. **2**: p. 69-72.
47. Fisher, J., et al., *Prevalence and determinants of common perinatal mental disorders in women in low- and lower-middle-income countries: a systematic review.* Bulletin of the World Health Organization, 2012. **90**(2): p. 139-149.
48. Rahman, A., Z. Iqbal, and R. Harrington, *Life events, social support and depression in childbirth: perspectives from a rural community in the developing world.* Psychological Medicine, 2003. **33**(7): p. 1161-1167.
49. Barber, J.A. and S.G. Thompson, *Analysis and interpretation of cost data in randomized controlled trials: review of published studies.* BMJ, 1998. **317**(7167): p. 1195-1200.
50. Efron, B. and R. Tibshirani, *An Introduction to the Bootstrap, vol. 23, no. 2.* 1998, Chapman & Hall, New York.
51. Fenwick, E., K. Claxton, and M. Sculpher, *Representing uncertainty: the role of cost-effectiveness acceptability curves.* Health economics, 2001. **10**(8): p. 779-787.
52. Hamdani, S.U., et al., *WHO Parents Skills Training (PST) programme for children with developmental disorders and delays delivered by Family Volunteers in rural Pakistan: study protocol for effectiveness implementation hybrid cluster randomized controlled trial.* Global mental health, 2017. **4**.

Acknowledgment

The study sponsor and funders have no role in the study design; collection, management, analysis, and interpretation of data; writing of the report; and the decision to submit the report for publication.

Authors' Contributors

SUH, ZeH & DW will have the access of all datasets. ZeH drafted the initial manuscript. SUH conceived the study and wrote the final manuscript. AG, FS, AR, BM, HJ, DW & AR contributed to the writing. All authors approved of the final version of the manuscript.

Funding statement

This work is supported by Grand Challenges Canada, Saving Brains, Grant Number SB-POC-1809-18680

Competing interests statement None declared.

Word count 5436 (without Tables and Figure)

For peer review only - http://bmjopen.bmj.com/site/about/guidelines.xhtml

Fig 1: Flow Diagram

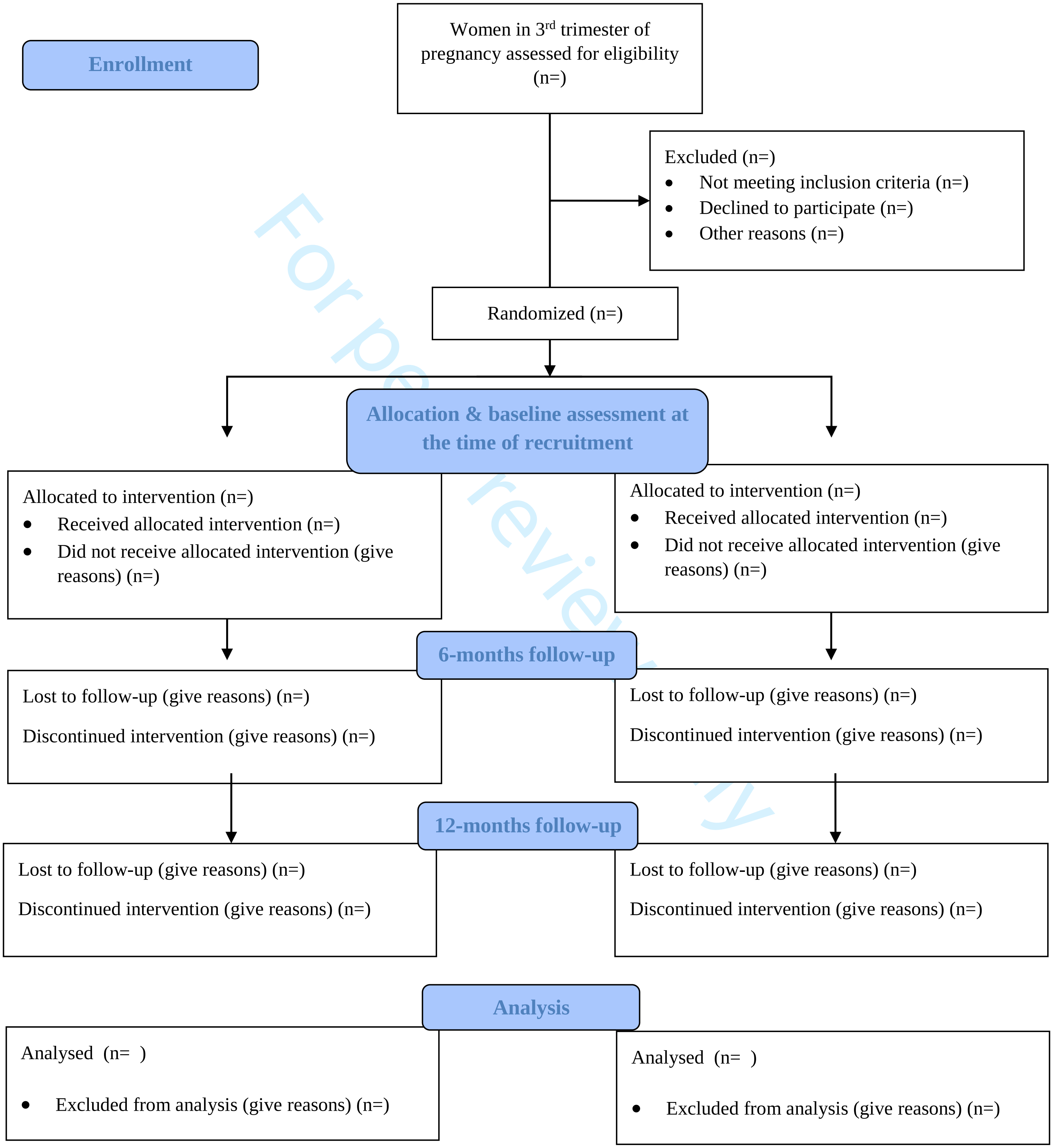

For peer review only - http://bmjopen.bmj.com/site/about/guidelines.xhtml

STANDARD PROTOCOL ITEMS: RECOMMENDATIONS FOR INTERVENTIONAL TRIALS

SPIRIT 2013 Checklist: Recommended items to address in a clinical trial protocol and related documents*

Section/item	Item No	Description	Addressed on page no.
**Administrative information**			
Title	1	Descriptive title identifying the study design, population, interventions, and, if applicable, trial acronym	Title p.1
Trial registration	2a	Trial identifier and registry name. If not yet registered, name of intended registry	2
	2b	All items from the World Health Organization Trial Registration Data Set	Included in the registration
Protocol version	3	Date and version identifier	N/A
Funding	4	Sources and types of financial, material, and other support	20
Roles and responsibilities	5a	Names, affiliations, and roles of protocol contributors	1 & 20
	5b	Name and contact information for the trial sponsor	N/A
	5c	Role of study sponsor and funders, if any, in study design; collection, management, analysis, and interpretation of data; writing of the report; and the decision to submit the report for publication, including whether they will have ultimate authority over any of these activities	20
	5d	Composition, roles, and responsibilities of the coordinating centre, steering committee, endpoint adjudication committee, data management team, and other individuals or groups overseeing the trial, if applicable (see Item 21a for data monitoring committee)	16
**Introduction**			
Background and rationale	6a	Description of research question and justification for undertaking the trial, including summary of relevant studies (published and unpublished) examining benefits and harms for each intervention	3

For peer review only

	6b	Explanation for choice of comparators	10
Objectives	7	Specific objectives or hypotheses	3
Trial design	8	Description of trial design including type of trial (eg, parallel group, crossover, factorial, single group), allocation ratio, and framework (eg, superiority, equivalence, noninferiority, exploratory)	3
**Methods: Participants, interventions, and outcomes**			
Study setting	9	Description of study settings (eg, community clinic, academic hospital) and list of countries where data will be collected. Reference to where list of study sites can be obtained	4
Eligibility criteria	10	Inclusion and exclusion criteria for participants. If applicable, eligibility criteria for study centres and individuals who will perform the interventions (eg, surgeons, psychotherapists)	4-5
Interventions	11a	Interventions for each group with sufficient detail to allow replication, including how and when they will be administered	6-10
	11b	Criteria for discontinuing or modifying allocated interventions for a given trial participant (eg, drug dose change in response to harms, participant request, or improving/worsening disease)	5
	11c	Strategies to improve adherence to intervention protocols, and any procedures for monitoring adherence (eg, drug tablet return, laboratory tests)	4
	11d	Relevant concomitant care and interventions that are permitted or prohibited during the trial	10
Outcomes	12	Primary, secondary, and other outcomes, including the specific measurement variable (eg, systolic blood pressure), analysis metric (eg, change from baseline, final value, time to event), method of aggregation (eg, median, proportion), and time point for each outcome. Explanation of the clinical relevance of chosen efficacy and harm outcomes is strongly recommended	10-13
Participant timeline	13	Time schedule of enrolment, interventions (including any run-ins and washouts), assessments, and visits for participants. A schematic diagram is highly recommended	13 & Fig 1 (submitted as a

For peer review only

		(see Figure)	pdf file)
Sample size	14	Estimated number of participants needed to achieve study objectives and how it was determined, including clinical and statistical assumptions supporting any sample size calculations	5
Recruitment	15	Strategies for achieving adequate participant enrolment to reach target sample size	4
**Methods: Assignment of interventions (for controlled trials)**			
Allocation:			
Sequence generation	16a	Method of generating the allocation sequence (eg, computer-generated random numbers), and list of any factors for stratification. To reduce predictability of a random sequence, details of any planned restriction (eg, blocking) should be provided in a separate document that is unavailable to those who enrol participants or assign interventions	13
Allocation concealment mechanism	16b	Mechanism of implementing the allocation sequence (eg, central telephone; sequentially numbered, opaque, sealed envelopes), describing any steps to conceal the sequence until interventions are assigned	13
Implementation	16c	Who will generate the allocation sequence, who will enrol participants, and who will assign participants to interventions	13
Blinding (masking)	17a	Who will be blinded after assignment to interventions (eg, trial participants, care providers, outcome assessors, data analysts), and how	13
	17b	If blinded, circumstances under which unblinding is permissible, and procedure for revealing a participant's allocated intervention during the trial	13
**Methods: Data collection, management, and analysis**			
Data collection methods	18a	Plans for assessment and collection of outcome, baseline, and other trial data, including any related processes to promote data quality (eg, duplicate measurements, training of assessors) and a description of study instruments (eg, questionnaires, laboratory tests) along with their reliability and validity, if known. Reference to where data collection forms can be found, if not in the protocol	10-13
	18b	Plans to promote participant retention and complete follow-up, including list of any outcome data to be collected for participants who discontinue or deviate from	4

For peer review only

		intervention protocols	
Data management	19	Plans for data entry, coding, security, and storage, including any related processes to promote data quality (eg, double data entry; range checks for data values). Reference to where details of data management procedures can be found, if not in the protocol	13
Statistical methods	20a	Statistical methods for analysing primary and secondary outcomes. Reference to where other details of the statistical analysis plan can be found, if not in the protocol	14
	20b	Methods for any additional analyses (eg, subgroup and adjusted analyses)	14-15
	20c	Definition of analysis population relating to protocol non-adherence (eg, as randomised analysis), and any statistical methods to handle missing data (eg, multiple imputation)	14
**Methods: Monitoring**			
Data monitoring	21a	Composition of data monitoring committee (DMC); summary of its role and reporting structure; statement of whether it is independent from the sponsor and competing interests; and reference to where further details about its charter can be found, if not in the protocol. Alternatively, an explanation of why a DMC is not needed	16
	21b	Description of any interim analyses and stopping guidelines, including who will have access to these interim results and make the final decision to terminate the trial	N/A
Harms	22	Plans for collecting, assessing, reporting, and managing solicited and spontaneously reported adverse events and other unintended effects of trial interventions or trial conduct	14
Auditing	23	Frequency and procedures for auditing trial conduct, if any, and whether the process will be independent from investigators and the sponsor	N/A
**Ethics and dissemination**			
Research ethics approval	24	Plans for seeking research ethics committee/institutional review board (REC/IRB) approval	13-14
Protocol amendments	25	Plans for communicating important protocol modifications (eg, changes to eligibility criteria, outcomes, analyses) to relevant parties (eg, investigators, REC/IRBs, trial participants, trial registries, journals, regulators)	N/A
Consent or assent	26a	Who will obtain informed consent or assent from potential trial participants or authorised surrogates, and how (see Item 32)	5

For peer review only

	26b	Additional consent provisions for collection and use of participant data and biological specimens in ancillary studies, if applicable	N/A
Confidentiality	27	How personal information about potential and enrolled participants will be collected, shared, and maintained in order to protect confidentiality before, during, and after the trial	14
Declaration of interests	28	Financial and other competing interests for principal investigators for the overall trial and each study site	20
Access to data	29	Statement of who will have access to the final trial dataset, and disclosure of contractual agreements that limit such access for investigators	20
Ancillary and post-trial care	30	Provisions, if any, for ancillary and post-trial care, and for compensation to those who suffer harm from trial participation	N/A
Dissemination policy	31a	Plans for investigators and sponsor to communicate trial results to participants, healthcare professionals, the public, and other relevant groups (eg, via publication, reporting in results databases, or other data sharing arrangements), including any publication restrictions	16-17
	31b	Authorship eligibility guidelines and any intended use of professional writers	N/A
	31c	Plans, if any, for granting public access to the full protocol, participant-level dataset, and statistical code	N/A
**Appendices**			
Informed consent materials	32	Model consent form and other related documentation given to participants and authorised surrogates	N/A
Biological specimens	33	Plans for collection, laboratory evaluation, and storage of biological specimens for genetic or molecular analysis in the current trial and for future use in ancillary studies, if applicable	N/A

*It is strongly recommended that this checklist be read in conjunction with the SPIRIT 2013 Explanation & Elaboration for important clarification on the items. Amendments to the protocol should be tracked and dated. The SPIRIT checklist is copyrighted by the SPIRIT Group under the Creative Commons "Attribution-NonCommercial-NoDerivs 3.0 Unported" license.

For peer review only - http://bmjopen.bmj.com/site/about/guidelines.xhtml
